# A Visible Light-Driven Minisci-Type Reaction with *N*-Hydroxyphthalimide Esters

**DOI:** 10.3390/molecules23040764

**Published:** 2018-03-27

**Authors:** Lisa Marie Kammer, Aliyaah Rahman, Till Opatz

**Affiliations:** Institute of Organic Chemistry, Johannes Gutenberg-University, 55128 Mainz, Germany; kammer@uni-mainz.de (L.M.K.); arahman@students.uni-mainz.de (A.R.)

**Keywords:** photoredox reactions, Minisci reaction, visible light, free radicals, heterocycles, photocatalysis

## Abstract

A visible light-promoted protocol for the redox-neutral coupling of *N*-hydroxyphthalimide esters with different *N*-heterocyclic compounds is described. The reaction proceeds through an alkyl radical intermediate generated by reductive decarboxylation of *N*-hydroxyphthalimide esters. In contrast to the original Minisci protocol, polyalkylation can largely be avoided. Mechanistic investigations revealed a radical chain mechanism which in some cases can proceed even if no photocatalyst is added. This valuable and functional group-tolerant reaction produces substituted heterocycles in moderate to excellent yield. The use of inexpensive starting materials and LEDs as the light source are key features of this C–C bond formation.

## 1. Introduction

**Scheme 1:**
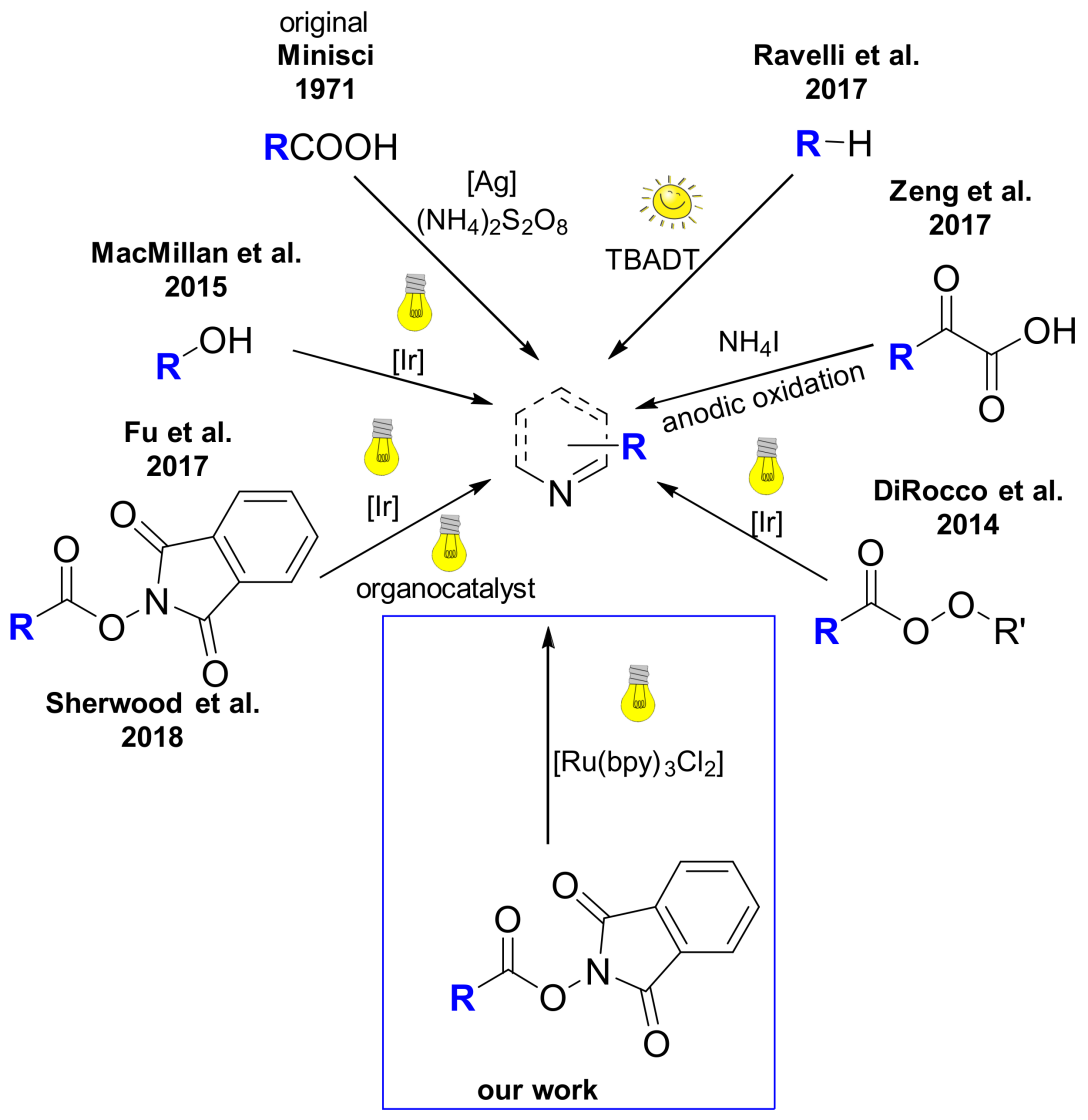
Different methods for alkylation of *N*-heteroarenes.

C–H functionalization is a popular synthetic strategy for the late-stage modification of heteroarenes [1,2,3]. Substituted heteroaromatics are common structural motifs in natural products as well as in synthetic pharmaceuticals [4,5,6]. Furthermore, they find application as ligands in metal catalysis [7,8,9]. Therefore, this compound class is of uncontested relevance for modern chemical and pharmaceutical research [4]. A particularly interesting option for the late-stage modification of heteroaromatic systems lies in their reaction with free radicals which can be generated under conditions tolerating a wide variety of functional groups [10]. Minisci and coworkers used the oxidative decarboxylation of carboxylic acids for the radical functionalization of a wide variety of heteroarenes [11,12]. The radical generation was effected by electron transfer from carboxylate ions to Ag^2+^ ions generated in situ from Ag^+^ through the action of peroxydisulfate as the terminal oxidant. The original experimental procedure used an excess of carboxylic acid and peroxydisulfate. These rather harsh reaction conditions limit the substrate scope as oxidation-sensitive moieties are incompatible with them. Furthermore, the alkylation of heteroarenes through alkyl radicals only slightly reduces their reactivity under these conditions and leads to polyalkylation of substrates having more than one reactive position. To overcome these problems, a number of variants of the original Minisci reaction have been developed over the past decades. For example, Zeng et al. reported the first electrochemical Minisci reaction with α-keto acids and ammonium iodide as a redox mediator (Scheme 1) [13]. In the course of the resurgence of photoredox catalysis, DiRocco et al. reported the first visible light-mediated Minisci reaction with acyl peroxides as precursors of alkyl radicals [14]. Ravelli and coworkers reported a sunlight-induced Minisci-type reaction with tetrabutylammonium decatungstate (TBADT) as an unusual photocatalyst [15]. Many publications in this direction have followed, in which different types of radical precursors and catalysts were employed. For example, primary alcohols or carboxylic acids, preactivated as their *N*-hydroxyphthalimide esters (NHP-esters), have been used as radical precursors by several groups; the latter were chosen to overcome the high and often limiting redox potential for the decarboxylation of their carboxylic acid precursors (vide infra) [9,16,17,18,19,20,21,22]. Fu reported a photoredox-catalyzed Minisci-type reaction with NHP-esters and an expensive iridium catalyst. After the experimental work of our article was completed, Sherwood and coworkers reported a one-pot photoredox Minisci reaction based on NHP-esters in which they used the non-commercial organocatalyst 1,2,3,5-tetrakis(carbazol-9-yl)-4,6-dicyanobenzene [23]. Here, we report a photoredox-catalyzed Minisci protocol based on NHP-esters and Ru(bpy)_3_Cl_2_ which is commercially available or can be prepared in a single step in 70% yield and does not represent a significant economic factor for work on laboratory scale [24]. In contrast to Sherwood’s protocol, the reactions presented here do not require heating and only lead to an insignificant degree of polysubstitution.

NHP-esters can be synthesized in a single step from carboxylic acids and can be converted to alkyl radicals by one-electron reduction and expulsion of CO_2_ and the phthalimide anion [21,25]. Carboxylic acids are attractive precursors since they are non-toxic, inexpensive, and are available in large structural diversity [26]. The redox potential of NHP-esters is significantly lower than that of the corresponding acid due to the weak N–O bond [21,25]. Here, we report a new method for a visible-light mediated Minisci-type reaction which is devoid of strong external oxidants. This method could overcome the above-mentioned limitations of the original Minisci protocol.

## 2. Results and Discussion

### 2.1. Method Development

For the decarboxylative alkylation, *N*-[(cyclohexylcarbonyl)oxy]phthalimide (**1**) and isoquinoline (**2**) were used as test substrates during the investigations of the reaction conditions. Trifluoroacetic acid was added to lower the LUMO coefficient of the heterocycle through protonation, thus facilitating the nucleophilic attack of the nucleophilic alkyl radical [10,11,12]. First, the catalytic activity of different photoredox catalysts under irradiation with LEDs was tested. While rhodamine 6 G and eosin Y provided a low conversion of the starting material, Ru(bpy)_3_Cl_2_ turned out to be the optimum catalyst in this screening, with a yield of 24% for **3** (Table 1, entry 4). The iridium catalyst shown in Table 1 provided the product in lower yield than did the ruthenium catalyst. The amount of Ru(bpy)_3_Cl_2_ can be reduced to 1 mol % without a loss in yield of **3** (see the Supporting Information, Table S1). The use of even lower amounts however resulted in reduced efficiency.

With solvent screening (Supporting Information, Table S2), the yield of model compound **3** could be increased up to 91% (2 mol % Ru(bpy)_3_Cl_2_) with DMF as the solvent of choice. In general, the reactions proceed better in polar solvents, while no conversion was detected in DCM. The reaction time was extended to 48 h to reach complete conversion of the starting heteroarene (as judged by TLC). In addition, different acidic additives were screened (Supporting Information, Table S3), including acetic acid, Lewis acids, and inorganic as well as stronger organic acids. The product was formed in high yields with different Lewis acids, concentrated hydrochloric acid, and sulfuric acid. A quantitative yield of the product after purification was only obtained with *p*-toluenesulfonic acid monohydrate (also using 2 mol % Ru(bpy)_3_Cl_2_). Acetic acid seems not to be acidic enough to promote the reaction [27]. It is also worth noting that the reaction proceeds with isoquinolinium triflate without further additive in a yield of 31% (Supporting Information, Table S3). A large excess of additive is not required, but superstoichiometric amounts (1.50 equiv.) provide a slightly higher yield than stoichiometric amounts. (Supporting Information, Table S3). Optimization of the starting material equivalents showed that a ratio of 1.50 equiv. *N*-hydroxyphthalimide ester to 1.00 equiv. of isoquinoline provides the highest yield (Supporting Information, Table S4). Control experiments verified that the reaction requires both light and an acidic additive to proceed (Table 2, entries 1 and 2). With 2,2,6,6-tetramethylpiperidinyloxyl (TEMPO) as a radical trap, intermediate **4** was intercepted and the respective trapping product could be detected via ESI-HRMS (Table 2, entry 6), which is a strong indication for a radical mechanism.

### 2.2. Mechanistic Considerations

**Scheme 2:**
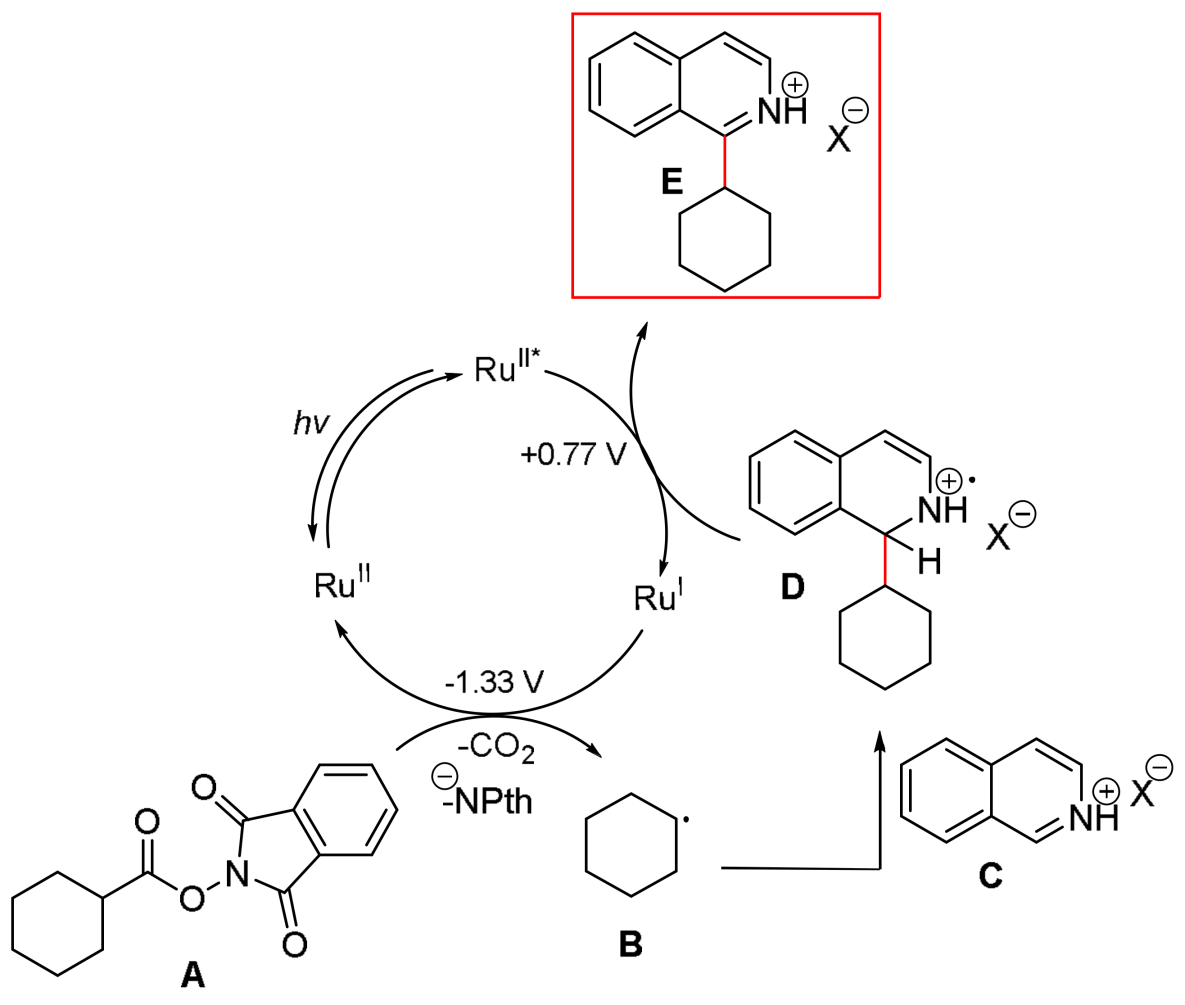
Proposed mechanism for the photoredox Minisci type reaction.

For the reaction with Ru(bpy)_3_^2+^, we propose a reductive decarboxylation of the NHP-ester **A** to generate the alkyl radical. Regarding the redox potentials of the catalyst (Ru^II*/I^ = +0.77 V, Ru^III/II*^ = −0.88 V, Ru^III/II^ = +1.29 V, Ru^II/I^ = −1.33 V vs. SCE), NHP-esters (E_1/2_ = −1.26 V to −1.37 V vs. SCE) are not able to oxidize excited Ru^II*^ to Ru^III^ [9,25]. Therefore, a reductive quenching cycle will have to proceed during the reaction, in which the NHP-ester **A** is reduced by a Ru^I^ species to produce the alkyl radical **B**. After addition of the radical to the protonated hetarene **C**, the resulting radical cation **D** is then oxidized under deprotonation to close the catalytic cycle (Scheme 2). In accordance with Fu, we suggest an off-cycle reaction as the initial step for the catalytic cycle to proceed [28]. Whether this is the oxidation of the solvent, the protonated arene, or the decomposition of the NHP-ester to the alkyl radical still needs to be investigated.

**Figure 1:**
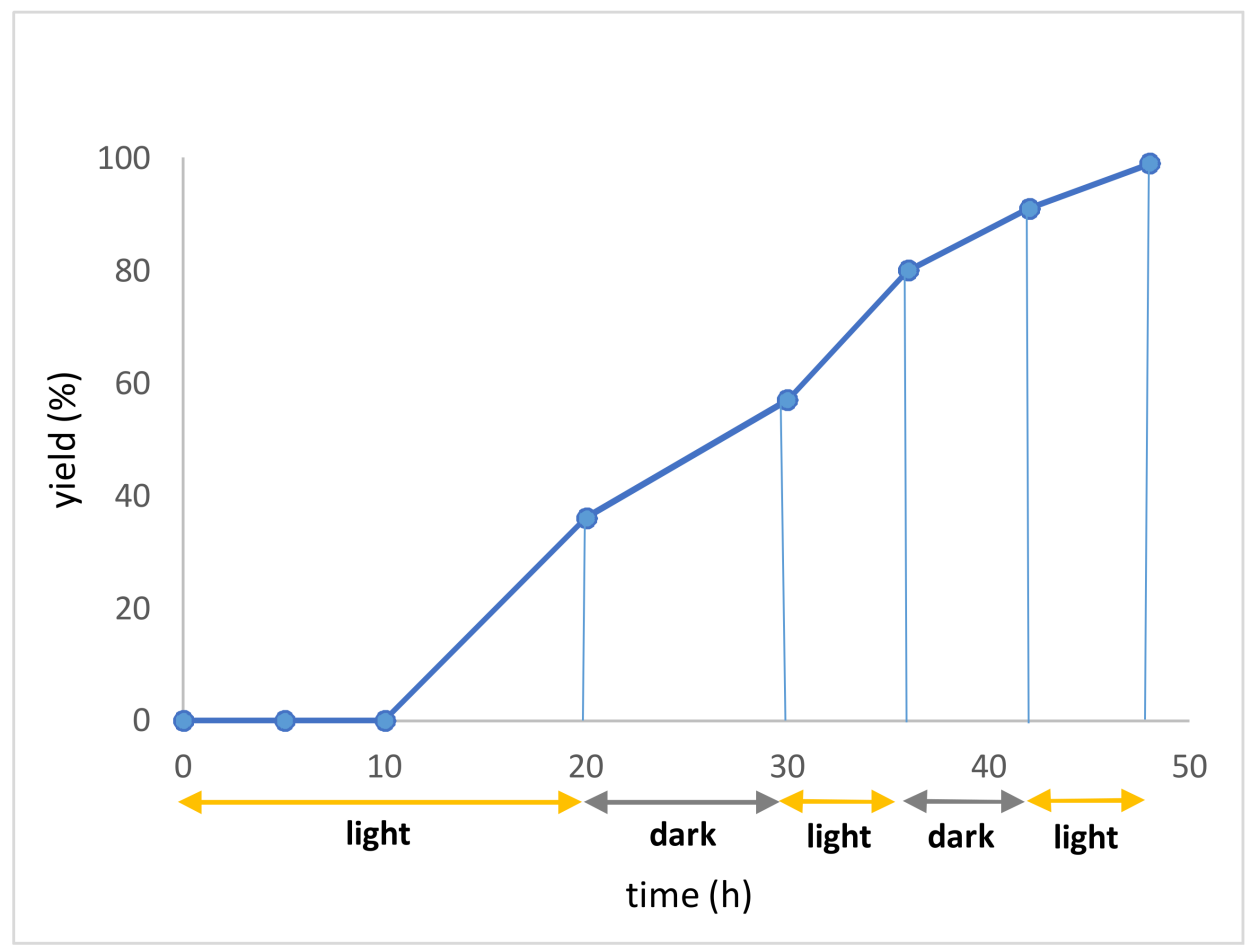
Light-dark cycle experiment with *N*-[(pentyl-2-carbonyl)oxy]phthalimide (0.3 mmol), isoquinoline (0.2 mmol) and *p*-toluenesulfonic acid (0.3 mmol) in DMF (0.1 M). The reaction mixture was irradiated for certain times with a 100 W blue LED module (emission spectra see Supporting Information, Figure S2) and the yield was determined by NMR spectroscopy with 1,4-bis-(trimethylsilyl)benzene as an internal standard.

While performing the control experiments, we observed that the reaction also proceeds without a catalyst under irradiation with visible light with certain substrates (Table 2, entry 3). Sherwood and coworkers have recently reported the same observation [17]. Our control experiments demonstrated that the reaction without the catalyst does not proceed at elevated temperatures in the dark (Table 2, entry 5). Adding TEMPO to the catalyst-free reaction mixture neither resulted in the formation of product nor trapping product **4** (Table 2, entry 4) and led to the suggestion of a free radical chain mechanism. UV-Vis measurements were performed to see if any individual compound or their combination in the reaction mixture absorbs light in the visible light region (see Supporting Information, Figures S3 and S4). None of them have any significant light absorption above 350 nm and the spectrum of the mixture is only the mere sum of the spectra of individual components. Thus, any further interactions, e.g., the formation of ground-state charge-transfer complexes, which may act as a photocatalyst, can be excluded and do not account for the surprising catalyst-independent reaction (see Supporting Information, Figure S4). To gain further insight into the mechanism, we performed a ‘light-dark cycle’ experiment (Figure 1). 

**Scheme 3:**
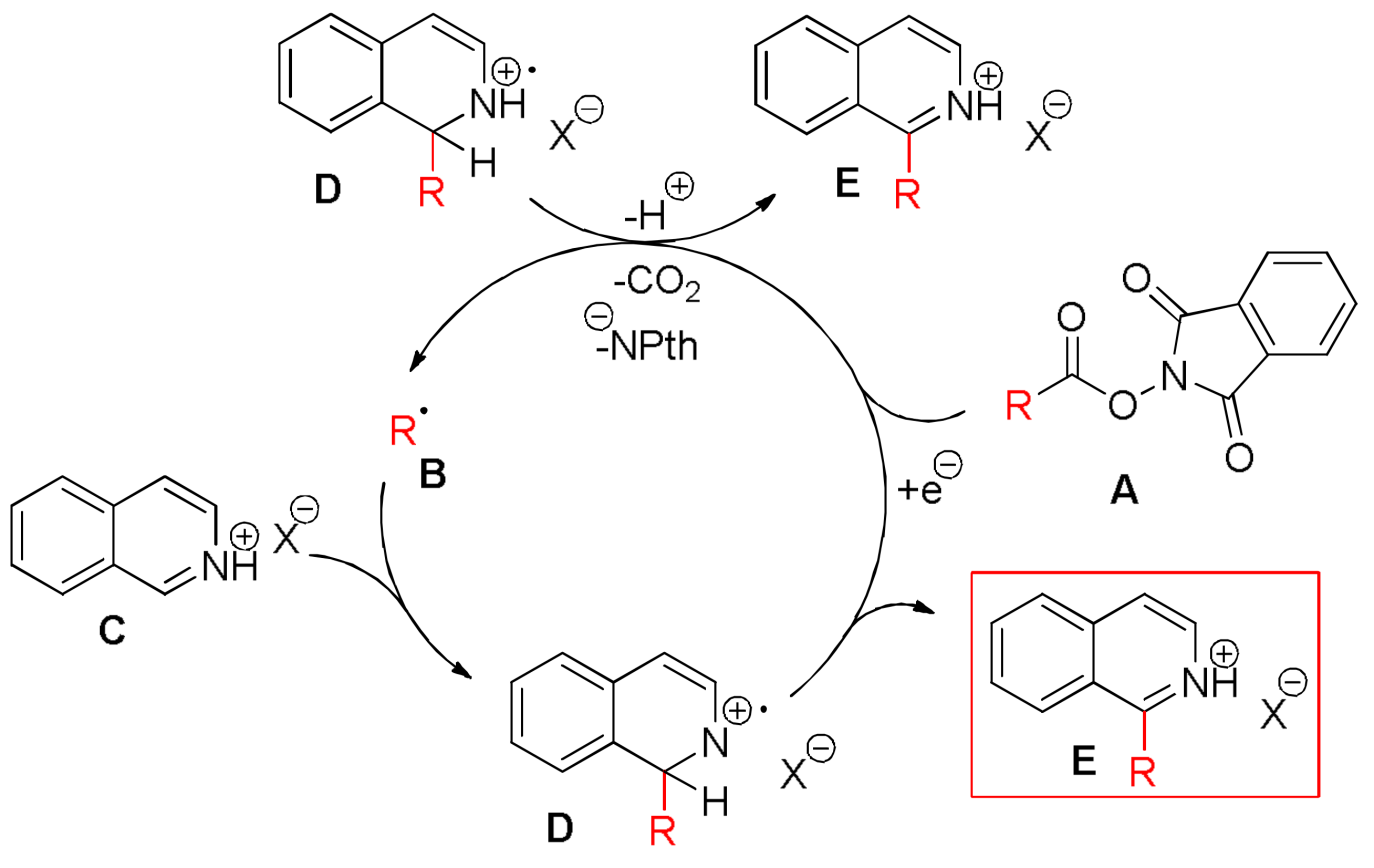
Proposed mechanism for the radical-chain reaction without a catalyst.

The diagram in Figure 1 clearly points towards a radical-chain mechanism, due to the continuing transformation during dark periods. Within the first 10 h of irradiation, no conversion of the starting material was detected and the reaction mixture was still colorless. After twenty hours of irradiation, the product was detected by NMR spectroscopy with an internal standard in 36% yield and the color of the mixture changed to light yellow. Henceforward, the reaction proceeded until complete consumption of the starting material within 48 hours. Color changing of the solution might be an indication for the start of the radical chain and the fact that the UV-Vis absorption spectrum of the yellow reaction mixture shows a weak absorption in the blue region of the visible light region could lead to the assumption that increased radical formation through increased light absorption could occur (see Supporting Information, Figure S3). Although the origin of the yellow color and its role for the reaction path at higher conversion are not yet understood, we propose the following radical-chain mechanism for the uncatalyzed reaction (Scheme 3). The time profile of the product formation in the light-dark cycle experiment does not provide evidence for a significant autocatalysis which would lead to an exponential curve—nevertheless, the induction period of the reaction remains to be investigated.

Through an off-cycle reaction, radical **B** is produced from NHP-ester **A**. After attack to isoquinolinium (**C**), radical cation **D** is generated which acts as a reductant for **A** to propagate the radical chain process.

### 2.3. Substrate Scope

**Scheme 4:**
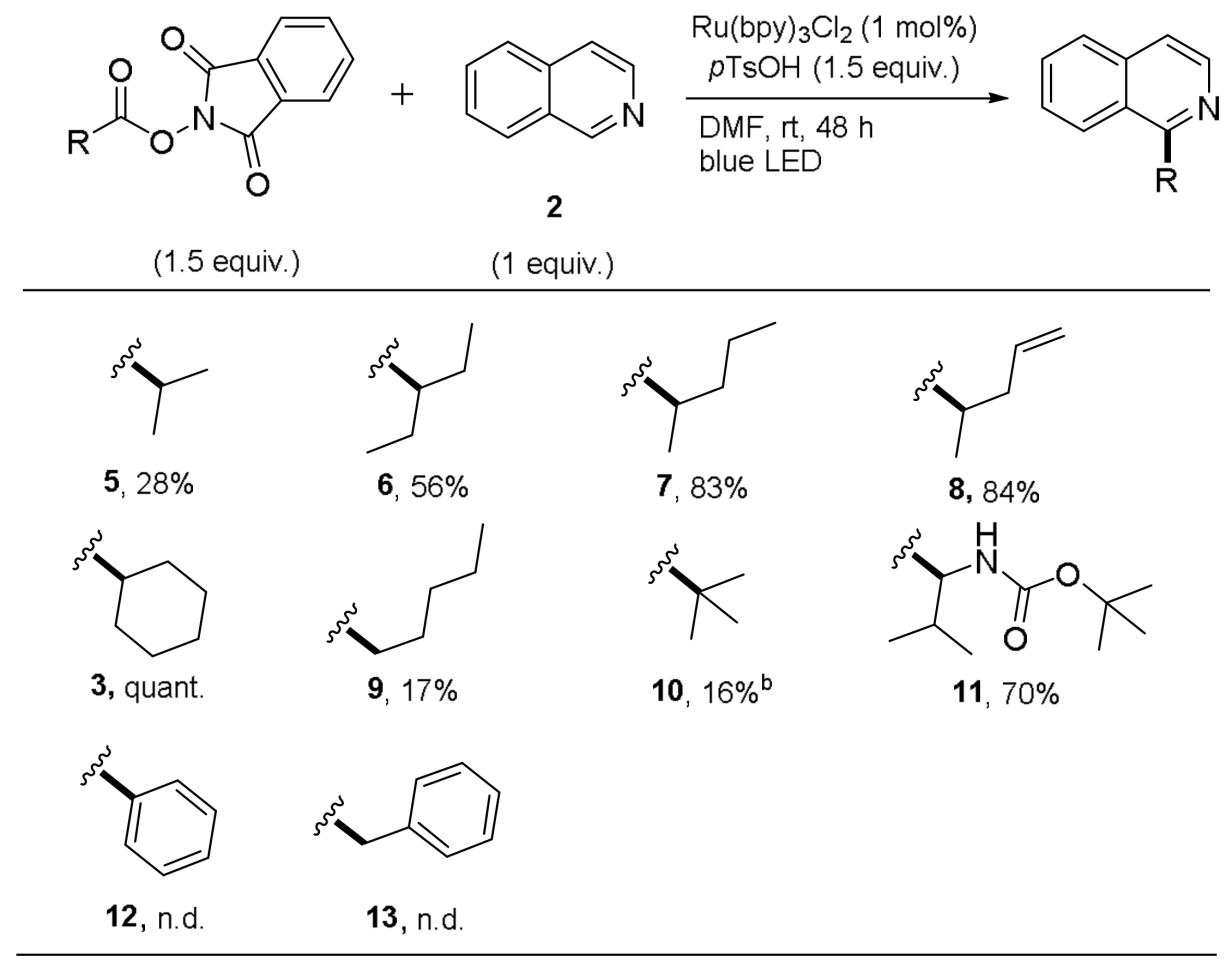
NHP-ester scope. ^b^ Yield determined by NMR with 1,4-bis-trimethylsilylbenzene as an internal standard. n.d. = not detected.

**Scheme 5:**
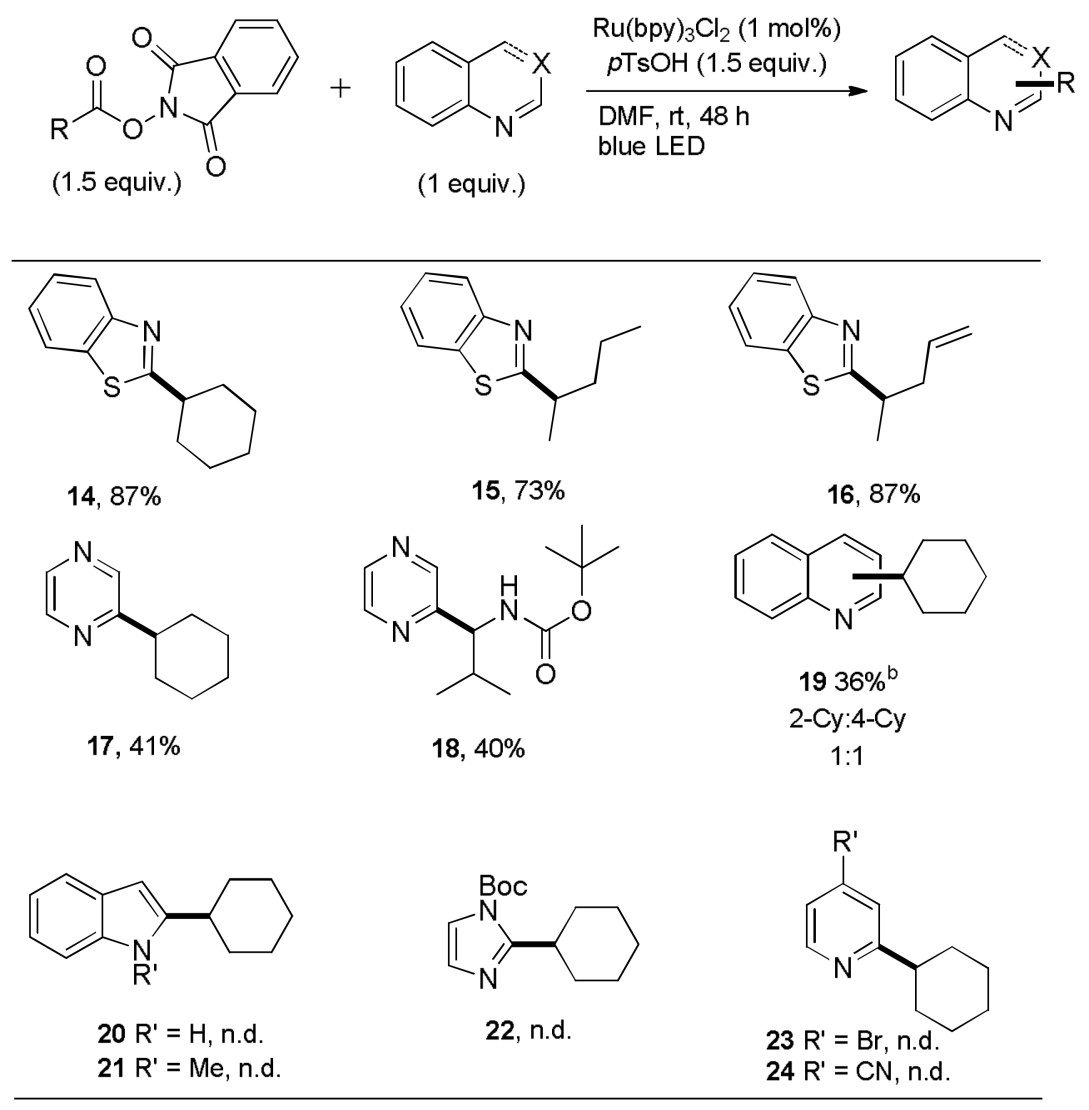
Heteroarene scope. ^b^ Ratio was determined by NMR with 1,4-bis-trimethylsilylbenzene as an internal standard. n.d. = not detected.

The synthesis of the starting material was performed according to a modified protocol of Overman (General Information A) [25]. Ten different NHP-esters were synthesized in 62% to quantitative yield and were subjected to the Minisci-type C–C coupling reaction using 1 mol % Ru(bpy)_3_Cl_2_. Small and unfunctionalized primary and secondary alkyl groups could be introduced in moderate to high yields (Scheme 4, **3**, **5**–**9**). Primary alkyl radicals yielded **9** in 17%, while a tertiary alkyl group was introduced in 16% (**10**). The best results were obtained for secondary alkyl groups. We generated five different secondary alkyl radicals and the products were formed in every case. While the cyclohexyl substituted product **3** was formed in quantitative yield, the isopropyl derivative **5** was obtained in only 28% yield. The other three secondary alkyl radicals formed the product in moderate to high yields (Scheme 4, **6** in 56%, **7** in 83%, **8** in 84%). Gratifyingly, α-amino acids furnished the desired coupling products in moderate to high yields (70% for **11** in Scheme 4, 40% for **18** in Scheme 5) and the common Boc-protecting group is tolerated [29]. Even the terminal double bond in compound **8** remained untouched during the reaction. *N*-(Benzoyloxy)phthalimide and *N*-(2-phenylacetyloxy)phthalimide proved to be unreactive, probably due to the unfavorable formation of an sp^2^-centered radical intermediate or, in the case of compound **13**, a highly stabilized and thus less reactive benzylic radical [30]. 

To determine the heteroarene scope, we explored 5- to 6-membered benzo-fused-/heterocycles regarding their electronic properties. Apart from isoquinoline (Scheme 4) we used benzothiazole, which provided compound **14**–**16** in high yields (Scheme 5). Pyrazine reacted smoothly to give compounds **17** in 41% and **18** in 40% yield. In the case of quinoline, a 1:1 ratio of 2- to 4-substituted product **19** was detected by NMR and the isomeric mixture was isolated in 36% yield. Gratifyingly, polyalkylation of quinoline was observed only in traces as judged by LC-MS and for pyrazine and isoquinoline no polyalkylation could be detected. Changing the heteroarene to electron rich systems such as indole or Boc-imidazole resulted in a complex reaction mixture and the desired alkylation products could not be detected. Similar results were obtained for 4-cyano-/4-bromopyridine. 

## 3. Materials and Methods

### 3.1. General Information 

Solvents and reagents were purchased from commercial suppliers and were used as received, unless otherwise stated. Tris-(2,2′-bipyridyl)ruthenium(II)chloride was synthesized according to a literature procedure [24]. Anhydrous THF was distilled and collected under an argon atmosphere from sodium and benzophenone. Reaction solvents were degassed in an ultrasonic bath by argon sparging for 20 min. Reactions requiring anhydrous conditions were performed in dried glassware under an atmosphere of argon. Flash column chromatography was carried out using silica gel (35–70 μm; ThermoFisher Acros Organics, Geel, Belgium). Thin-layer chromatography (TLC) was carried out on Merck silica gel plates (60 F_254_) using defined solvent mixtures; plates were visualized under UV light and/or by using TLC staining reagents. Melting points were determined in open capillary tubes using a digital electrothermal apparatus (Krüss, Hamburg, Germany). IR spectra were measured with a Bruker Tensor (Billerica, MA, USA) 27 instrument with a diamond ATR unit, and are reported in terms of frequency of absorption ($\tilde{\nu }$ [cm^−1^]). NMR spectra were recorded with Bruker 300 MHz (300 MHz for ^1^H, and 75.4 MHz for ^13^C) and 400 MHz (400 MHz for ^1^H, and 100.6 MHz for ^13^C) spectrometers with 5 mm BBFO probe heads. Residual solvent signals from the deuterated solvents were used as an internal reference (CDCl_3_: δ_H_ = 7.26 ppm, δ_C_ = 77.16 ppm) and reported in parts per million (ppm) relative to trimethylsilane (TMS) [31]. Coupling constants (*J*) are given in Hz using the conventional abbreviations (br = broad, s = singlet, d = doublet, t = triplet, q = quartet, sept = septet, m = multiplet and combinations thereof). Electron spray ionization (ESI) mass spectra were recorded on an Agilent (Waldbronn, Germany) 1200 series HPLC system with binary pump and integrated diode array detector coupled to an Agilent ion trap mass spectrometer or on an Agilent ESI quadrupole instrument. ESI-HRMS spectra were recorded with a Q-TOF instrument with a dual source and a suitable external calibrant. Elemental analysis was performed with an Elementar vario EL cube. Substances were decomposed under a stream of helium and the percentage amount of C, H, N, and S were recorded. Irradiation was carried out using a blue LED (high power 100 W LED, HPR40E-48K100BG (GalnN/GaN) from Huey Jann Electronic Industry Co., Ltd., Taichung City, Taiwan, *λ*_max_ = 462 ± 3 nm).

### 3.2. General Procedure A: Steglich Esterification of Carboxylic Acids with N-Hydroxyphthalimide ***1***, ***26***–***34***

A 100 mL Schlenk flask equipped with a stir bar and a septum was flushed with argon and charged with dry THF (30 mL). The carboxylic acid (1.00 equiv.), *N*,*N’*-dicyclohexylcarbodiimide (1.20 equiv.), 4-dimethylaminopyridine (0.10 equiv.) were added. After stirring for one minute, *N*-hydroxyphthalimide (1.10 equiv.) was added and the reaction mixture was stirred for 24 h at room temperature. The precipitating dicyclohexyl urea was filtered off and the solution was concentrated by evaporation of the solvent. Flash column chromatography afforded the desired product.

*N-(Cyclohexylcarbonyloxy)phthalimide* (**1**). This compound was prepared according to General Procedure A using cyclohexanecarboxylic acid (1.00 g, 7.80 mmol). Purifications of the crude product by flash column chromatography (SiO_2_, ^*c*^Hex/EtOAc 5/1) afforded the title compound (2.12 g, 7.74 mmol, 99%) as a colorless solid. *R*_f_ = 0.38 (SiO_2_, ^*c*^Hex/EtOAc 5/1). IR (ATR): $\tilde{\nu }$ (cm^−1^) 2935, 2858, 1786, 1742, 1372, 1186, 1142, 1001, 697. ^1^H-NMR, COSY (400 MHz, CDCl_3_): δ/ppm 7.89–7.86 (m, 2H, H-3,6), 7.79–7.77 (m, 2H, H-4,5), 2.73 (tt, *J* = 11.0, 3.7 Hz, 1H, H-1′), 2.12–2.07 (m, 2H, H’), 1.85–1.80 (m, 2H, H’), 1.69–1.63 (m, 4H, H-2′,6′), 1.42–1.31 (m, 2H, H4′). ^13^C, HSQC, HMBC (100.6 MHz, CDCl_3_): δ/ppm 171.9 (CO), 163.2 (2C, CO), 134.8 (2C, C-4,5), 129.1 (2C, C_*q*_), 124.0 (2C, C-3,6), 40.6 (CH), 28.9 (CH_2_), 25.6 (CH_2_), 25.2 (CH_2_). Mp: 69.1–70.3 °C. FD-MS: *m*/*z* 273.0 (100%, [M]^+^). EA: anal. C 65.84, H 5.61, N 5.26%, calcd for C_15_H_15_NO_4_, C 65.92, H 5.53, N 5.13%.

*N-(Hexanoyloxy)phthalimide* (**26**). This compound was prepared according to General Procedure A using hexanoic acid (1.00 g, 8.60 mmol). Purifications of the crude product by flash column chromatography (SiO_2_, ^*c*^Hex/EtOAc 5/1) afforded the title compound (1.89 g, 7.23 mmol, 84%) as a colorless solid. *R*_f_ = 0.32 (SiO_2_, ^*c*^Hex/EtOAc 10/1). IR (ATR): $\tilde{\nu }$ [cm^−1^] 2957, 1816, 1788, 1740, 1467, 1186, 1065, 878, 670. ^1^H-NMR, COSY (400 MHz, CDCl_3_): δ/ppm 7.90–7.86 (m, 2H, H-3,6), 7.81–7.76 (m, 2H, H-4,5), 2.68 (t, *J* = 7.4 Hz, 2H, H-2′), 1.82 (q, *J* = 7.6 Hz, 2H, H-3′), 1.48–1.40 (m, 4H, H-4′,5′), 0.95 (t, *J* = 7.4 Hz, 3H, H-6′). ^13^C, HSQC, HMBC (100.6 MHz, CDCl_3_): δ/ppm 169.8 (CO), 162.1 (2C, CO), 134.8 (2C, C-4,5), 129.1 (2C, C_*q*_), 139.9 (2C, C-3,6), 31.0 (C-2′,4′), 24.4 (C-3′), 22.2 (C-5′), 13.8 (C-6′). Mp: 41.2–42.9 °C. FD-MS: *m*/*z* 261.0 (100%, [M]^+^). EA: anal. C 64.46, H 5.57, N 5.51%, calcd for C_14_H_15_NO_4_, C 64.36, H 5.79, N 5.36%.

*N-(Pivaloyloxy)phthalimide* (**27**). This compound was prepared according to General Procedure A using pivalic acid (1.00 g, 9.79 mmol). Purifications of the crude product by flash column chromatography (SiO_2_, ^*c*^Hex/EtOAc 5/1) afforded the title compound (2.40 g, 9.70 mmol, 99%) as a colorless solid. *R*_f_ = 0.61 (SiO_2_, ^*c*^Hex/EtOAc 3/1). IR (ATR): $\tilde{\nu }$ [cm^−1^] 2936, 1784, 1743, 1486, 1369, 1186, 1062, 1023, 878,697. ^1^H-NMR, COSY (300 MHz, CDCl_3_): δ/ppm 7.86–7.84 (m, 2H, H-3,6), 7.77–7.74 (m, 2H, H-4,5), 1.41 (s, 9H, C(CH_3_)_3_). ^13^C, HSQC, HMBC (75.4 MHz, CDCl_3_): δ/ppm 174.5(CO), 162.2 (2C, CO), 134.8 (2C, C-4,5), 129.1 (2C, C_*q*_), 123.9 (2C, C-2,6), 38.5 (C_*q*_), 27.1 (CH_3_). Mp: 76.5–79.3 °C. FD-MS: *m*/*z* 247.4 (100%, [M]^+^). The spectral data are consistent with those reported in the literature [32].

*N-(N-tert-Butoxycarbonyl*-l-*valinoyloxy)phthalimide* (**28**). This compound was prepared according to General Procedure A using *N*-(*tert*-butoxycarbonyl)-l-valine (1.50 g, 6.90 mmol). Purifications of the crude product by flash column chromatography (SiO_2_, ^*c*^Hex/EtOAc 9/1) afforded the title compound (1.71 g, 4.72 mmol, 68%) as a colorless solid. *R*_f_ = 0.17 (SiO_2_, ^*c*^Hex/EtOAc 9/1). IR (ATR): $\tilde{\nu }$ [cm^−1^] 2973, 1788, 1742, 1708, 1366, 1184, 1136, 1069, 876, 696. ^1^H-NMR, COSY (400 MHz, CDCl_3_): δ/ppm 7.89 (dd, *J* = 5.5, 3.1 Hz, 2H, H-3,6), 7.80 (dd, *J* = 5.5, 3.1 Hz, 2H, H-4,5), 5.04 (d, *J* = 9.0 Hz, 1H, NH), 4.67 (dd, *J* = 9.0, 4.8 Hz, 1H, C*H), 2.28–2.34 (m, 1H, C*H*(CH_3_)_2_), 1.47 (s, 9H, (CH_3_)_3_), 1.14 (s, 3H, CH_3_), 1.10 (s, 3H, CH_3_). ^13^C, HSQC, HMBC (100.6 MHz, CDCl_3_): δ/ppm 169.9 (CO), 161.7 (2C, CO), 155.3 (CO), 134.8 (2C, C-4,5), 129.0 (2C, C_*q*_), 124.0 (2C, C-3,6), 80.6 (C_*q*_(CH_3_), 57.0 (C*H), 31.8 (*C*H(CH_3_)_2_), 28.2 (3C, C_*q*_(*C*H_3_)_3_), 18.7 (CH_3_), 17.3 (CH_3_). Mp: 79.6–80.9 °C. ESI-MS (pos): *m*/*z* 363.1 (100%, [M + H]^+^). ESI-HRMS (pos): *m*/*z* calculated for [C_18_H_14_N_2_O_6_]^+^ *m*/*z* 363.1551, found 363.1567. 

*N-(Isobutyryloxy)phthalimide* (**29**). This compound was prepared according to General Procedure A using isobutyric acid (1.00 g, 11.4 mmol). Purifications of the crude product by flash column chromatography (SiO_2_, ^*c*^Hex/EtOAc 5/1) afforded the title compound (2.48 g, 10.6 mmol, 93%) as a colorless solid. *R*_f_ = 0.52 (SiO_2_, ^*c*^Hex/EtOAc 2/1). IR (ATR): $\tilde{\nu }$ [cm^−1^] 2879, 1845, 1811, 1739, 1467, 1359, 1048, 967, 877, 739. ^1^H-NMR, COSY (300 MHz, CDCl_3_): δ/ppm 7.89–7.86 (m, 2H, H-3,6), 7.79–7.76 (m, 2H, H-4,5), 2.95 (sept, *J* = 7.0 Hz, 1H, C*H*), 1.38 (s, 3H, C*H*_3_). 1.36 (s, 3H, C*H*_3_). ^13^C, HSQC, HMBC (75.4 MHz, CDCl_3_): δ/ppm 173.2 (CO), 162.2 (2C, CO), 134.8 (2C, C-4,5), 129.1 (2C, C_*q*_), 124.0 (2C, C-3,6), 31.9 (CH), 19.0 (2C, CH_3_). Mp: 66.4–67.7 °C. FD-MS: *m*/*z* 233.4 (100%, [M]^+^). The spectral data are consistent with those reported in the literature [33].

*N-(2-Methylpentanoyloxy)phthalimide* (**30**). This compound was prepared according to General Procedure A using 2-methylpentanoic acid (1.00 g, 8.60 mmol). Purifications of the crude product by flash column chromatography (SiO_2_, ^*c*^Hex/EtOAc 5/1) afforded the title compound (1.48 g, 5.36 mmol, 62%) as a colorless solid. *R*_f_ = 0.57 (SiO_2_, ^*c*^Hex/EtOAc 3/1). IR (ATR): $\tilde{\nu }$ [cm^−1^] 2978, 2875, 1844, 1783, 1481, 1062, 1023, 878, 805, 696. Mp: 29.8–31.2 °C. ^1^H-NMR, COSY (300 MHz, CDCl_3_): δ/ppm 7.87–7.84 (m, 2H, H-3,6), 7.78–7.75 (m, 2H, H-4,5), 2.87–2.82 (m, 1H, H-2′), 1.84–1.80 (m, 1H, H-3a’), 1.64–1.56 (m, 1H, H-3b’), 1.54–1.44 (m, 2H, H-4′), 1.33 (d, *J* = 7.0 Hz, 3H, CHC*H*_3_), 0.96 (t, *J* = 7.1 Hz, 3H, H-5′). ^13^C, HSQC, HMBC (75.4 MHz, CDCl_3_): δ/ppm 172.9 (CO), 162.1 (2C, CO), 134.8 (2C, C-4,5), 129.1 (2C, C_*q*_), 124.0 (2C, C-3,6), 37.0 (C-2′), 35.9 (C-3′), 20.2 (C-4′), 17.0 (CH*C*H_3_), 14.0 (C-5′). FD-MS: *m*/*z* 261.3 (100%, [M]^+^). EA: anal. C 64.12, H 5.78, N 5.66%, calcd for C_14_H_15_NO_4_, C 64.36, H 5.79, N 5.36%.

*N-(2-Ethylbutyryloxy)phthalimide* (**31**). This compound was prepared according to General Procedure A using 2-ethylbutyric acid (1.00 g, 8.60 mmol). Purifications of the crude product by flash column chromatography (SiO_2_, ^*c*^Hex/EtOAc 5/1) afforded the title compound (1.48 g, 5.36 mmol, 62%) as a colorless oil. *R*_f_ = 0.58 (SiO_2_, ^*c*^Hex/EtOAc 3/1). IR (ATR): $\tilde{\nu }$ [cm^−1^] 2968, 2879, 1811, 1742, 1466, 1369, 1135, 967, 677, 696. ^1^H-NMR, COSY (300 MHz, CDCl_3_): δ/ppm 7.86–7.83 (m, 2H, H-3,6), 7.77–7.74 (m, 2H, H-4,5), 2.58 (tt, *J* = 8.6, 5.5 Hz, 1H, H-3′), 1.78–1.71 (m, 4H, H-2′,4′), 1.05 (t, *J* = 7.5 Hz, 6H, H-1′,5′). ^13^C, HSQC, HMBC (75.4 MHz, CDCl_3_): δ/ppm 172.3 (CO), 162.1 (2C, CO), 134.8 (2C, C-4,5), 129.0 (2C, C_*q*_), 123.9 (2C, C-3,6), 46.5 (C-3′), 25.3 (C-2′,4′), 11.6 (C-1′,5′). FD-MS: *m*/*z* 261.4 (100%, [M]^+^). EA: anal. C 63.98, H 5.93, N 5.35%, calcd for C_14_H_15_NO_4_, C 64.36, H 5.79, N 5.36%.

*N-(Pent-4-enyl-2-carbonyloxy)phthalimide* (**32**). This compound was prepared according to General Procedure A using 2-methyl-4-pentenoic acid (1.00 g, 8.76 mmol). Purifications of the crude product by flash column chromatography (SiO_2_, ^*c*^Hex/EtOAc 9/1) afforded the title compound (2.16 g, 8.34 mmol, 95%) as a colorless oil. *R*_f_ = 0.37 (SiO_2_, ^*c*^Hex/EtOAc 8/1). IR (ATR): $\tilde{\nu }$ [cm^−1^] 2938, 1785, 1739, 1459, 1325, 1135, 1081, 967, 924, 696. ^1^H-NMR, COSY (300 MHz, CDCl_3_): δ/ppm 7.87–7.82 (m, 2H, H-3,6), 7.79–7.74 (m, 2H, H-4,5), 5.90–5.80 (m, 1H, H-4′), 5.20–5.11 (m, 2H, H-5′), 2.91 (sept, *J* = 6.9 Hz, 1H, H-2′), 2.57 (app dtt, *J* = 14.5, 6.9, 1.2 Hz, 1H, H-3a’), 2.37 (m, 1H, H-3b’), 1.34 (d, *J* = 6.9 Hz, 3H, H-5′). ^13^C, HSQC, HMBC (75.4 MHz, CDCl_3_): δ/ppm 172.1 (CO), 162.0 (2C, CO), 134.8 (2C, C-4,5), 134.0 (C-4′), 129.0 (2C, C_*q*_), 124.0 (2C, C-3,6), 118.3 (C-5′), 37.5 (C-2′), 36.9 (C-3′), 16.4 (C-1′). ESI-MS (pos): *m*/*z* 260.1 (100%, [M + H]^+^), 282.1 (54%, [M + Na]^+^). ESI-HRMS (pos): *m*/*z* calculated for [C_14_H_14_NO_4_]^+^ *m*/*z* 260.0917, found 260.0917.

*N-(Benzoyloxy)phthalimide* (**33**). This compound was prepared according to General Procedure A using benzoic acid (0.50 g, 4.09 mmol). Purifications of the crude product by flash column chromatography (SiO_2_, ^*c*^Hex/EtOAc 3/1) afforded the title compound (1.01 g, 3.80 mmol, 93%) as a colorless solid. *R*_f_ = 0.29 (SiO_2_, ^*c*^Hex/EtOAc 3/1). IR (ATR): $\tilde{\nu }$ [cm^−1^] 1771, 1741, 1600, 1453, 1372, 1236, 1186, 1003, 877, 659. ^1^H-NMR, COSY (300 MHz, CDCl_3_): δ/ppm 8.21–8.18 (m, 2H, H-2′,6′), 7.94–7.92 (m, 2H, H-3,6), 7.83–7.80 (m, 2H, H-4,5), 7.70 (pseudo tt, *J* = 7.7, 1.2 Hz, 1H, H-4′), 7.57–7.51 (m, 2H, H-3′,5′). ^13^C, HSQC, HMBC (75.4 MHz, CDCl_3_): δ/ppm 162.9 (CO), 162.2 (2C, CO), 135.0 (C-4′), 134.9 (2C, C-4,5), 130.8 (2C, C-2′,6′), 129.2 (2C, C_*q*_), 129.0 (2C, C-3′,5′), 125.4 (C_*q*_), 124.2 (2C, C-3,6). Mp: 174.3–176.9 °C. FD-MS: *m*/*z* 267.0 (100%, [M]^+^). The spectral data are consistent with those reported in the literature [34].

*N-(2-Phenylacetyloxy)phthalimide* (**34**). This compound was prepared according to General Procedure A using phenylacetic acid (0.25 g, 1.84 mmol). Purifications of the crude product by flash column chromatography (SiO_2_, ^*c*^Hex/EtOAc 3/1) afforded the title compound (408 mg, 1.45 mmol, 79%) as a colorless solid. *R*_f_ = 0.36 (SiO_2_, ^*c*^Hex/EtOAc 3/1). IR (ATR): $\tilde{\nu }$ [cm^−1^] 1815, 1788, 1742, 1498, 1359, 1133, 1065, 878, 728, 519. ^1^H-NMR, COSY (300 MHz, CDCl_3_): δ/ppm 7.89–7.84 (m, 2H, H-3,6), 7.80–7.76 (m, 2H, H-4,5), 7.40–7.38 (m, 3H, H-2′,4′,6′), 7.36–7.31 (m, 2H, H-3′,5′), 4.00 (s, 2H, CH_2_). ^13^C, HSQC, HMBC (75.4 MHz, CDCl_3_): δ/ppm 167.9 (CO), 161.9 (2C, CO), 134.9 (2C, C-4,5), 131.6 (C_*q*_), 129.4 (2C, C-2′,6′), 129.0 (4C, C_*q*_, C-3′,5′), 127.9 (C-4′), 124.1 (2C, C-3,6), 37.8 (CH_2_). Mp: 174.8–178.0 °C. FD-MS: *m*/*z* 281.4 (100%, [M]^+^). The spectral data are consistent with those reported in the literature [35].

### 3.3. General Procedure B: Coupling of N-Hydroxyphthalimide Esters with Heterocycles ***3***, ***5***–***18***

A 10 mL vial equipped with a stirrer bar and a septum was flushed with argon and charged with dry *N*,*N*’-dimethylformamid. The solvent was previously degassed by ultrasonication (see Gerneral Information). The *N*-hydroxyphthalimide ester (0.30 mmol, 1.50 equiv.), the heterocyclic compound (0.20 mmol, 1 equiv.), *p*-toluenesulfonic acid monohydrate (0.30 mmol, 1.50 equiv.) and Ru(bpy)_3_Cl_2_ (2 μmol, 1 mol %) were added, followed by argon sparging for 1 min. The mixture was irradiated with a blue LED module from a distance of 5 cm for 48 h. A saturated aqueous NaHCO_3_ solution (30 mL) was added, followed by extraction with dichloromethane (3 × 15 mL). The combined organic layers were dried over MgSO_4_ and concentrated under reduced pressure. Flash column chromatography afforded the desired product.

*1-(Cyclohexyl)isoquinoline* (**3**). This compound was prepared according to General Procedure B using *N*-(cyclohexycarbonyloxy)phthalimide (82.00 mg, 0.30 mmol) and Isoquinoline (25.8 mg, 0.20 mmol). Purifications of the crude product by flash column chromatography (SiO_2_, ^*c*^Hex/EtOAc 14/1) afforded the title compound (42.30 mg, 0.2 mmol, quant.) as a yellow oil. *R*_f_ = 0.32 (SiO_2_, ^*c*^Hex/EtOAc 14/1). IR (ATR): $\tilde{\nu }$ [cm^−1^] 2926, 2852, 1562, 1449, 1391, 1335, 993, 822, 744, 675. ^1^H-NMR, COSY (300 MHz, CDCl_3_): δ/ppm 8.48 (d, *J* = 5.6 Hz, 1H, H-3), 8.22 (d, *J* = 8.1 Hz, 1H, H-8), 7.84–7.81 (m, 1H, H-5), 7.64 (mc, 1H, H-6), 7.58 (ddd, *J* = 8.1, 6.8, 1.3 Hz, 1H, H-7), 7.48 (d, *J* = 5.6, 0.8 Hz, 1H, H-4), 3.56 (tt, *J* = 11.6, 3.3 Hz, 1H, H-1′), 2.00–1.93 (m, 4H, H’), 1.88–1.80 (m, 4H, H‘), 1.58–1.33 (m, 2H, H-4′). ^13^C, HSQC, HMBC (75.4 MHz, CDCl_3_): δ/ppm 165.7 (C_*q*_-1), 141.9 (C-3), 136.4 (C_*q*_-4a), 129.5 (C-6), 127.5 (C-5), 126.3 (2C, C-7,C_*q*_-8a), 124.7 (C-8), 118.9 (C-4), 41.5 (C-1′), 32.6 (2C, C-3′,5′), 26.9 (2C, C-2′,6′), 26.3 (C-4′). ESI-MS (pos): *m*/*z* 212.1 (100%, [M + H]^+^). The spectral data are consistent with those reported in the literature [36].

*1-(Propan-2-yl)isoquinoline* (**5**). This compound was prepared according to General Procedure B using *N*-(isobutyryloxy)phthalimide (70.00 mg, 0.30 mmol) and isoquinoline (25.8 mg, 0.20 mmol). Purifications of the crude product by flash column chromatography (SiO_2_, ^*c*^Hex/EtOAc 10/1) afforded the title compound (9.50 mg, 5.55 × 10^−5^ mol, 28%) as a colorless oil. *R*_f_ = 0.44 (SiO_2_, ^*c*^Hex/EtOAc 8/1). IR (ATR): $\tilde{\nu }$ [cm^−1^] 3631, 1719, 1655, 1586, 1543, 1008, 903,869, 822, 727. ^1^H-NMR, COSY (300 MHz, CDCl_3_): δ/ppm 8.49 (d, *J* = 5.7 Hz, 1H, H-3), 8.23 (app dq, *J* = 8.3, 0.8 Hz, 1H, H-8), 7.83–7.80 (m, 1H, H-5), 7.62 (m, 2H, H-6,7), 7.49 (dd, *J* = 5.7, 0.8 Hz, 1H, H-4), 3.96 (sept, *J* = 6.9 Hz, 1H, H-2′), 1.46 (s, 3H, CH_3_), 1.44 (s, 3H, CH_3_). ^13^C, HSQC, HMBC (75.4 MHz, CDCl_3_): δ/ppm 166.5 (C_*q*_-1), 142.0 (C-3), 136.5 (C_*q*_-4a), 129.7 (C-6), 127.7 (C-5), 127.0 (C-7), 126.4 (C_*q*_-8a), 124.9 (C-8), 119.1 (C-4), 31.1 (C-2′), 22.4 (2C, CH_3_). ESI-MS (pos): *m*/*z* 172.1 (100%, [M + H]^+^). The spectral data are consistent with those reported in the literature [37].

*1-(Pentan-3-yl)isoquinoline* (**6**). This compound was prepared according to General Procedure B using *N*-(2-ethylbutyryloxy)phthalimide (52.3 mg, 0.30 mmol) and isoquinoline (25.8 mg, 0.20 mmol). Purifications of the crude product by flash column chromatography (SiO_2_, ^*c*^Hex/EtOAc 8/1) afforded the title compound (22.3 mg, 1.12 × 10^−4^ mol, 56%) as a colorless oil. *R*_f_ = 0.47 (SiO_2_, ^*c*^Hex/EtOAc 8/1). IR (ATR): $\tilde{\nu }$ [cm^−1^] 3050, 2960, 2929, 2872, 1585, 1458, 1313, 821, 745, 682. ^1^H-NMR, COSY (300 MHz, CDCl_3_): δ/ppm 8.53 (d, *J* = 5.7 Hz, 1H, H-3), 8.25 (ddd, *J* = 8.4, 1.4, 0.7 Hz, 1H, H-8), 7.83–7.80 (m, 1H, H-5), 7.62 (mc, 2H, H-6,7), 7.48 (dd, *J* = 5.7, 0.9 Hz, 1H, H-4), 3.54 (tt, *J* = 8.3, 5.5 Hz, 1H, H-3′), 1.98–1.80 (m, 4H, H-2′,4′), 0.79 (t, *J* = 7.4 Hz, 6H, H-1′,5′). ^13^C, HSQC, HMBC (75.4 MHz, CDCl_3_): δ/ppm 165.2 (C_*q*_-1), 142.2 (C-3), 136.4 (C_*q*_-4a), 129.6 (C-6), 128.3 (C_*q*_-8a), 127.6 (C-5), 126.9 (C-7), 125.1 (C-8), 118.9 (C-4), 44.9 (C-3′), 28.3 (2C, C-2′,4′), 12.5 (C-1′,5′). ESI-MS (pos): *m*/*z* 200.1 (100%, [M + H]^+^). The spectral data are consistent with those reported in the literature [38]. 

*1-(Pentan-2-yl)isoquinoline* (**7**). This compound was prepared according to General Procedure B using *N*-(2-methylpentanoyloxy)phthalimide (78.40 mg, 0.30 mmol) and isoquinoline (25.8 mg, 0.20 mmol). Purifications of the crude product by flash column chromatography (SiO_2_, ^*c*^Hex/EtOAc 8/1) afforded the title compound (33.1 mg, 1.66 × 10^−4^ mol, 83%) as a colorless oil. *R*_f_ = 0.37 (SiO_2_, ^*c*^Hex/EtOAc 8/1). IR (ATR): $\tilde{\nu }$ [cm^−1^] 3050, 2957, 2929, 2870, 1585, 1456, 1309, 821, 745, 683. ^1^H-NMR, COSY (300 MHz, CDCl_3_): δ/ppm 8.50 (d, *J* = 5.7 Hz, 1H, H-3), 8.23 (app dq, *J* = 8.0, 0.8 Hz, 1H, H-8), 7.83–7.80 (m, 1H, H-5), 7.62 (mc, 2H, H-6,7), 7.48 (dd, *J* = 5.7, 0.8 Hz, 1H, H-4), 3.82 (sept, *J* = 6.9 Hz, 1H, H-2′), 2.01–1.94 (m, 1H, H-3′), 1.75–1.67 (m, 1H, H-3′), 1.41 (d, *J* = 6.9 Hz, 3H, H-1′), 1.36–1.25 (m, 2H, H-4′), 0.90 (t, *J* = 7.3 Hz, 3H, H-5′). ^13^C, HSQC, HMBC (75.4 MHz, CDCl_3_): δ/ppm 166.2 (C_*q*_-1), 142.1 (C-3), 136.5 (C_*q*_-4a), 129.7 (C-6), 127.7 (C-5), 127.0 (C-7), 126.9 (C_*q*_-8a), 124.9 (C-8), 118.9 (C-4), 39.1 (C-3′), 35.0 (C-2′), 21.1 (C-4′), 20.7 (C-1′), 14.4 (C-5′). ESI-MS (pos): *m*/*z* 200.1 (100%, [M + H]^+^). The spectral data are consistent with those reported in the literature [37]. 

*1-(Pent-4-en-2-yl)isoquinoline* (**8**). This compound was prepared according to General Procedure B using *N*-(pent-4-enyl-2-carbonyloxy)phthalimide (77.7 mg, 0.30 mmol) and isoquinoline (25.8 mg, 0.20 mmol). Purifications of the crude product by flash column chromatography (SiO_2_, ^*c*^Hex/EtOAc 8/1) afforded the title compound (33.1 mg, 1.68 × 10^−4^ mol, 84%) as a yellow oil. *R*_f_ = 0.47 (SiO_2_, ^*c*^Hex/EtOAc 8/1). IR (ATR): $\tilde{\nu }$ [cm^−1^] 3052, 2969, 2928, 1639, 1622, 1374, 994, 913, 822, 798. ^1^H-NMR, COSY (300 MHz, CDCl_3_): δ/ppm 8.50 (d, *J* = 5.8 Hz, 1H, H-3), 8.23–8.20 (m, 1H, H-8), 7.83–7.80 (m, 1H, H-5), 7.62 (mc, 2H, H-6,7), 7.49 (dd, *J* = 5.8, 0.9 Hz, 1H, H-4), 5.83 (ddd, *J* = 17.1, 10.2, 7.7 Hz, 1H, H-4′), 5.08–5.02 (m, 2H, H-5′), 3.87 (sept, *J* = 6.8 Hz, 1H, H-2′), 2.79–2.74 (m, 1H, H-3′), 2.50 (mc, 1H, H-3′), 1.43 (d, *J* = 6.8 Hz, 1H, H-1′). ^13^C, HSQC, HMBC (75.4 MHz, CDCl_3_): δ/ppm 165.2 (C_*q*_-1), 142.0 (C-3), 137.4 (C-4′), 136.5 (C_*q*_-4a), 129.7 (C-6), 127.7 (C-5), 127.0 (C-7), 126.7 (C_*q*_-8a), 124.8 (C-8), 119.1 (C-4), 116.6 (C-5′), 40.9 (C-3′), 36.2 (C-2′), 20.3 (C-1′). ESI-MS (pos): *m*/*z* 198.1 (100%, [M + H]^+^). ESI-HRMS (pos): *m*/*z* calculated for [C_14_H_15_N]^+^ *m*/*z* 198.1277, found 198.1278. 

*1-(Pentan-1-yl)isoquinoline* (**9**). This compound was prepared according to General Procedure B using *N*-(hexanoyloxy)phthalimide (78.4 mg, 0.30 mmol) and isoquinoline (25.8 mg, 0.20 mmol). Purifications of the crude product by flash column chromatography (SiO_2_, ^*c*^Hex/EtOAc 15/1) afforded the title compound (6.6 mg, 3.31 × 10^−5^ mol, 17%) as a yellow oil. *R*_f_ = 0.35 (SiO_2_, ^*c*^Hex/EtOAc 5/1). IR (ATR): $\tilde{\nu }$ [cm^−1^] 2926, 2852, 1562, 1449, 1391, 1335, 993, 822, 744, 675. ^1^H-NMR, COSY (300 MHz, CDCl_3_): δ/ppm 8.43 (d, *J* = 5.8 Hz, 1H, H-3), 8.16 (d, *J* = 8.5, 1.1 Hz, 1H, H-8), 7.83–7.80 (m, 1H, H-5), 7.63 (mc, 2H, H-6,7), 7.50 (dd, *J* = 5.8, 0.9 Hz, 1H, H-4), 3.32–3.26 (m, 2H, H-1′), 1.92–1.84 (m, 2H, H-2′), 1.50–1.40 (m, 2H, H-3‘,4′), 0.92 (t, *J* = 7.1 Hz, 3H, H-5′). ^13^C, HSQC, HMBC (75.4 MHz, CDCl_3_): δ/ppm 162.2 (C_*q*_-1), 142.2 (C-3), 136.4 (C_*q*_-4a), 129.9 (C-6), 127.5 (C-5), 127.1 (C-7), 125.5 (C-8), 124.1 (C_*q*_-8a), 119.3 (C-4), 35.7 (C-1′), 32.3 (C-3′), 29.7 (C-2′), 22.8 (C-4′), 14.2 (C-5′). ESI-MS (pos): *m*/*z* 200.1 (100%, [M + H]^+^). The spectral data are consistent with those reported in the literature [37].

*tert-Butyl-[1-(isoquinolin-2-yl)-2-methylpropyl]carbamate* (**11**). This compound was prepared according to General Procedure B using *N*-(*N*-*tert*-butoxycarbonyl-l-valinyloxy)phthalimide (108.7 mg, 0.30 mmol) and isoquinoline (25.8 mg, 0.20 mmol). Purifications of the crude product by flash column chromatography (SiO_2_, ^*c*^Hex/EtOAc 8/1) afforded the title compound (41.9 mg, 1.39 × 10^−4^ mol, 70%) as a colorless solid. *R*_f_ = 0.31 (SiO_2_, ^*c*^Hex/EtOAc 8/1). Mp: 108.3–109.9 °C. IR (ATR): $\tilde{\nu }$ [cm^−1^] 3631, 2969, 2931, 1718, 1508, 1459, 1170, 903, 727, 649. ^1^H-NMR, COSY (300 MHz, CDCl_3_): δ/ppm 8.46 (d, *J* = 5.7 Hz, 1H, H-3), 8.24 (d, *J* = 8.3 Hz, 1H, H-8), 7.84–7.81 (m, 1H, H-5), 7.65 (mc, 2H, H-6,7), 7.55 (dd, *J* = 5.7, 0.9 Hz, 1H, H-4), 6.07 (d, *J* = 9.1 Hz, 1H, NH), 5.54 (dd, *J* = 9.1, 5.7 Hz, 1H, H-2′), 2.25–2.16 (m, 1H, H-3′), 1.44 (s, 9H, C(C*H*_3_)_3_), 0.96 (d, *J* = 6.8 Hz, 1H, C*H*_3_), 0.87 (d, *J* = 6.8 Hz, 1H, C*H*_3_). ^13^C, HSQC, HMBC (75.4 MHz, CDCl_3_): δ/ppm 160.4 (C_*q*_-1), 156.2 (CO), 141.4 (C-3), 136.4 (C_*q*_-4a), 130.1 (C-6), 127.5 (2C, C-5,7), 126.2 (C_*q*_-8a), 125.0 (C-8), 120.1 (C-4), 79.2 (C_*q*_(CH_3_)_3_), 55.6 (C-2′), 34.9 (*C*H(CH_3_)_2_), 28.6 (3C, (*C*H_3_)_3_), 20.5 (*C*H_3_), 17.6 (*C*H_3_) . ESI-MS (pos): *m*/*z* 301.2 (100%, [M + H]^+^). ESI-HRMS (pos): *m*/*z* calculated for [C_18_H_24_N_2_O_2_]^+^ *m*/*z* 301.1911, found 301.1909.

*2-Cyclohexyl-1,3-benzothiazole* (**14**). This compound was prepared according to General Procedure B using *N*-(cyclohexycarbonyloxy)phthalimide (82.0 mg, 0.30 mmol) and 1,3-benzothiazole (27.0 mg, 0.20 mmol). Purifications of the crude product by flash column chromatography (SiO_2_, ^*c*^Hex/EtOAc 8/1) afforded the title compound (37.8 mg, 1.74 × 10^−4^ mol, 87%) as a colorless oil. *R*_f_ = 0.48 (SiO_2_, ^*c*^Hex/EtOAc 8/1). IR (ATR): $\tilde{\nu }$ [cm^−1^] 2926, 2851, 1514, 1448, 1437, 1244, 1014, 757, 728, 668. ^1^H-NMR, COSY (300 MHz, CDCl_3_): δ/ppm 7.97 (dt, *J* = 8.2, 1.0 Hz, 1H, H-4), 7.86–7.83 (m, 1H, H-7), 7.44 (ddd, *J* = 8.3, 7.2, 1.3 Hz, 1H, H-5), 7.33 (ddd, *J* = 8.3, 7.2, 1.3 Hz, 1H, H-6), 3.10 (tt, *J* = 11.6, 3.6 Hz, 1H, H-1′), 2.23–2.18 (m, 2H, H-2′), 1.88–1.85 (m, 2H, H-3′), 1.76–1.67 (m, 3H, H-4′,6′), 1.52–1.38 (m, 3H, H-4′,5′). ^13^C, HSQC, HMBC (75.4 MHz, CDCl_3_): δ/ppm 177.7 (C_*q*_-2), 153.2 (C_*q*_-3a), 134.2 (C_*q*_-7a), 125.9 (C-5), 124.6 (C-6), 122.7 (C-4), 121.7 (C-7), 43.6 (C-1′), 33.6 (2C, C-2′,6′), 26.2 (2C, C-3′,5′), 25.7 (C-4′). ESI-MS (pos): *m*/*z* 218.0 (100%, [M + H]^+^). The spectral data are consistent with those reported in the literature [39].

*2-(Pentan-2-yl)-1,3-benzothiazole* (**15**). This compound was prepared according to General Procedure B using *N*-(2-methylbutyryloxy)phthalimide (78.4 mg, 0.30 mmol) and 1,3-benzothiazole (27.0 mg, 0.20 mmol). Purifications of the crude product by flash column chromatography (SiO_2_, ^*c*^Hex/EtOAc 40/1) afforded the title compound (29.8 mg, 1.45 × 10^−4^ mol, 73%) as a colorless oil. *R*_f_ = 0.39 (SiO_2_, ^*c*^Hex/EtOAc 30/1). IR (ATR): $\tilde{\nu }$ [cm^−1^] 2933, 1786, 1741, 1516, 1466, 1263, 1157, 1069, 1026, 877. ^1^H-NMR, COSY (300 MHz, CDCl_3_): δ/ppm 7.98 (ddd, *J* = 8.1, 1.3, 0.6 Hz, 1H, H-4), 7.85 (ddd, *J* = 7.9, 1.3, 0.6 Hz, 1H, H-7), 7.44 (ddd, *J* = 8.1, 7.3, 1.3 Hz, 1H, H-5), 7.33 (ddd, *J* = 7.9, 7.3, 1.3 Hz, 1H, H-6), 3.30 (sept, *J* = 7.0 Hz, 1H, H-1′), 1.91–1.81 (m, 1H, H-3′), 1.77–1.69 (m, 1H, H-3′), 1.45 (d, *J* = 6.9 Hz, 3H, H-1′), 1.39–1.34 (m, 2H, H-4′), 0.93 (t, *J* = 7.3 Hz, 3H, H-5′). ^13^C, HSQC, HMBC (75.4 MHz, CDCl_3_): δ/ppm 178.3 (C_*q*_-2), 153.2 (C_*q*_-3a), 134.2 (C_*q*_-7a), 125.9 (C-5), 124.7 (C-6), 122.7 (C-4), 121.7 (C-7), 40.0 (C-3′), 39.4 (C-2′), 21.3 (C-1′), 20.7 (C-4′), 14.1 (C-5′). ESI-MS (pos): *m*/*z* 206.0 (100%, [M + H]^+^). The spectral data are consistent with those reported in the literature [40].

*2-(Pent-4-en-2-yl)-1,3-benzothiazole* (**16**). This compound was prepared according to General Procedure B using *N*-(pent-4-enyl-2-carbonyloxy)phthalimide (77.7 mg, 0.30 mmol) and 1,3-benzothiazole (27.0 mg, 0.20 mmol). Purifications of the crude product by flash column chromatography (SiO_2_, ^*c*^Hex/EtOAc 40/1) afforded the title compound (35.4 mg, 1.74 × 10^−4^ mol, 87%) as a colorless oil. *R*_f_ = 0.52 (SiO_2_, ^*c*^Hex/EtOAc 10/1). IR (ATR): $\tilde{\nu }$ [cm^−1^] 3073, 2972, 2928, 2870, 1517, 1455, 1437, 916, 758, 729. ^1^H-NMR, COSY (300 MHz, CDCl_3_): δ/ppm 7.99 (ddd, *J* = 8.1, 1.2, 0.6 Hz, 1H, H-4), 7.85 (ddd, *J* = 7.9, 1.3, 0.7 Hz, 1H, H-7), 7.45 (ddd, *J* = 8.1, 7.2, 1.3 Hz, 1H, H-5), 7.34 (ddd, *J* = 7.9, 7.2, 1.3 Hz, 1H, H-6), 5.81 (dddd, *J* = 16.9, 10.1, 7.4, 6.6 Hz, 1H, H-4′), 5.12–5.07 (m, 2H, H-5′), 3.38 (sept, *J* = 7.0 Hz, 1H, H-2′), 2.69 (app dtt, *J* = 14.6, 6.6, 1.3 Hz, 1H, H-3′), 2.48 (app dtt, *J* = 14.6, 6.6, 1.3 Hz, 1H, H-3′), 1.47 (d, *J* = 6.9 Hz, 3H, H-1′). ^13^C, HSQC, HMBC (75.4 MHz, CDCl_3_): δ/ppm 177.1 (C_*q*_-2), 153.2 (C_*q*_-3a), 135.5 (C-4′), 134.8 (C_*q*_-7a), 126.0 (C-5), 124.8 (C-6), 122.8 (C-4), 121.7 (C-7), 117.5 (C-5′), 41.7 (C-3′), 39.3 (C-2′), 20.6 (C-1′). ESI-MS (pos): *m*/*z* 204.0 (100%, [M + H]^+^). ESI-HRMS (pos): *m*/*z* calculated for [C_12_H_13_NS]^+^ *m*/*z* 204.0841, found 204.0839.

*2-Cyclohexyl-pyrazine* (**17**). This compound was prepared according to General Procedure B using *N*-(cyclohexycarbonyloxy)phthalimide (82.0 mg, 0.30 mmol) and pyrazine (16.2 mg, 0.20 mmol). Purifications of the crude product by flash column chromatography (SiO_2_, ^*c*^Hex/EtOAc 5/1) afforded the title compound (13.2 mg, 8.14 × 10^−5^ mol, 41%) as a colorless oil. *R*_f_ = 0.30 (SiO_2_, ^*c*^Hex/EtOAc 5/1). IR (ATR): $\tilde{\nu }$ [cm^−1^] 3052, 2926, 2852, 1736, 1468, 1449, 1184, 1060, 1016, 839. ^1^H-NMR, COSY (300 MHz, CDCl_3_): δ/ppm 8.48–8.39 (m, 2H, H-5,6), 7.88–7.77 (m, 1H, H-3), 2.74 (tt, *J* = 12.1, 3.4 Hz, 1H, H-1′), 1.97–1.86 (m, 4H, H-2′,6′), 1.59–1.31 (m, 6H, H-3′,4′,5′). ^13^C, HSQC, HMBC (75.4 MHz, CDCl_3_): δ/ppm 161.7 (C_*q*_-1), 143.9 (CH), 143.6 (CH), 134.3 (C-3), 44.1 (C-1′), 32.5 (C-2′,6′), 26.4 (C-3′,5′), 25.9 (C-4′). ESI-MS (pos): *m*/*z* 163.0 (100%, [M + H]^+^). The spectral data are consistent with those reported in the literature [37].

*tert-Butyl-[2-methyl-1-(pyrazin-2-yl)propyl]carbamate* (**18**). This compound was prepared according to General Procedure B using *N*-(*N*-*tert*-butoxycarbonyl-l-valinyloxy)phthalimide (108.7 mg, 0.30 mmol) and pyrazine (16.2 mg, 0.20 mmol). Purifications of the crude product by flash column chromatography (SiO_2_, ^*c*^Hex/EtOAc 5/1) afforded the title compound (20.3 mg, 8.1 × 10^−5^ mol, 40%) as a colorless oil. *R*_f_ = 0.14 (SiO_2_, ^*c*^Hex/EtOAc 3/1). IR (ATR): $\tilde{\nu }$ [cm^−1^] 3057, 2968, 2931, 1702, 1522, 1275, 1171, 1017, 744, 667, 548. ^1^H-NMR, COSY (300 MHz, CDCl_3_): δ/ppm 8.52–8.51 (m, 2H, H-5,6), 8.47 (d, *J* = 2.4 Hz, 1H, H-3), 5.50 (d, *J* = 8.9 Hz, 1H, NH), 4.64 (t, *J* = 7.8 Hz, 1H, H-1′), 2.11 (sept, *J* = 6.8 Hz, 1H, H-2′), 1.43 (s, 9H, (C*H*_3_)_3_), 0.94 (d, *J* = 6.8 Hz, 1H, C*H*_3_), 0.87 (d, *J* = 6.8 Hz, 1H, C*H*_3_). ^13^C, HSQC, HMBC (75.4 MHz, CDCl_3_): δ/ppm 156.0 (CO), 155.7 (C_*q*_-2), 144.1 (2C, C-5,6), 143.4 (C-3), 79.7 (C_*q*_(CH_3_)_3_), 58.5 (C-1′), 33.9 (C-2′), 28.5 (3C, (*C*H_3_)_3_), 19.3 (*C*H_3_), 18.3 (*C*H_3_). ESI-MS (pos): *m*/*z* 252.1 (100%, [M + H]^+^), 274.1 (17%, [M + Na]^+^). ESI-HRMS (pos): *m*/*z* calculated for [C_13_H_21_N_3_O_2_]^+^ *m*/*z* 252.1703, found 252.1707.

## 4. Conclusions

In summary we reported a mild, visible light-mediated and Ru(bpy)_3_Cl_2_ catalyzed C–C alkylation reaction. Here, the starting material can be synthesized easily in one step with high yield from cheap, commercially available carboxylic acids. The problem of the original Minisci reaction regarding the formation of polyalkylated byproducts can be circumvented with our protocol [11]. With 1 mol % of the metal catalyst, the costs are decreased to the minimum and the catalyst enables radical generation without any further external oxidant. Furthermore, mechanistic studies gave us insight in the proceeding mechanism without any photocatalyst.

## Figures and Tables

**Table 1:** Catalyst screening. ^a^ A 100 W blue LED module was used for irradiation; ^b^ A 100 W green LED module was used for irradiation; ^c^ Detected via HPLC; ^d^ Isolated yield.

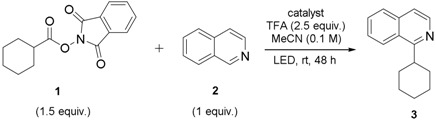		
**Entry**	**Catalyst (Loading in %)**	**Yield (%)**
1	rhodamine G (10 mol %) ^a^	traces ^c^
2	eosin Y (10 mol %) ^b^	traces ^c^
3	Ir(dFCF_3_ppy)_2_(dtbpy)^3+^ (2 mol %) ^a^	16% ^d^
**4**	**Ru(bpy)_3_Cl_2_ (2 mol %) ^a^**	**24% ^d^**

**Table 2:** Control experiments.

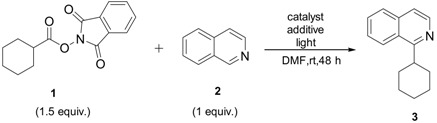		
**Entry**	**Parameter**	**Yield (%)**
1	no additive	/
2	no light	/
3	no catalyst	81% ^b^
4	no catalyst, TEMPO added (1.50 equiv.) ^a^	/
5	no catalyst, no light 45 °C	/
6	TEMPO added (1.50 equiv.) ^a^	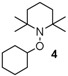 HRMS calc. for [C_15_H_30_O]^+^ = 240.2322, found = 240.2328

^a^ Unless stated otherwise, all reactions were performed according to general procedure B; ^b^ Isolated yield.

## Supplementary Materials

The following are available online, Figure S1: Setup for irradiation with a 100 W blue LED module. Figure S2: Emission spectra of the 100 W blue LED module. The spectra was recorded with an USB2000+ from Ocean Optics, Inc., Dunedin, FL, USA. Wavelength range from 200–523 nm, optical resolution 1 nm. Table S1: Catalyst loading studies. Table S2. Solvent screening for the photoredox Minisci-type reaction. Table S3. Additive screening for the photoredox Minisci-type reaction. Table S4. Equivalent screening of isoquinoline. Figure S3. UV-Vis absorption spectra at 290–500 nm measured for A) *N*-(cyclohexylcarbonyloxy)phthalimid (1 × 10^−4^ M), B) isoquinoline (1 × 10^−4^ M), *p*-toluenesulfonic acid (1 × 10^−4^ M), D) the reaction mixture (1 × 10^−4^ M), E) reaction mixture after product formation (yellowish solution 1 × 10^−4^ M) and E.1) UV-Vis absorption spectra at 390–500 nm of the reaction mixture after product formation (0.1 M). All spectra were measured in dry DMF. Figure S4: Exclusion of charge-transfer complexes. UV-Vis absorption spectra at 290–500 nm measured for different mixtures of *N*-(cyclohexylcarbonyloxy)phthalimid (1 × 10^−4^ M), B) isoquinoline (1 × 10^−4^ M), *p*-toluenesulfonic acid (1 × 10^−4^ M) and D) the reaction mixture (1 × 10^−4^ M). All spectra were measured in dry DMF. Figures S5–S39: NMR Spectra of compounds **1**, **3**, **5**–**11**, **14**–**19**, **26**–**34**.

## References

[1] Li J.J. (2015). C-H Bond Activation in Organic Synthesis.

[2] Hartwig J.F. (2008). Carbon–heteroatom bond formation catalysed by organometallic complexes. Nature.

[3] Lyons T.W., Sanford M.S. (2010). Palladium-Catalyzed Ligand-Directed C−H Functionalization Reactions. Chem. Rev..

[4] Duncton M.A.J. (2011). Minisci reactions: Versatile CH-functionalizations for medicinal chemists. MedChemComm.

[5] Cragg G.M., Newman D.J., Snader K.M. (1997). Natural Products in Drug Discovery and Development. J. Nat. Prod..

[6] Newman D.J., Cragg G.M. (2007). Natural Products as Sources of New Drugs over the Last 25 Years. J. Nat. Prod..

[7] Evans D.A., Woerpel K.A., Hinman M.M., Faul M.M. (1991). Bis(oxazolines) as chiral ligands in metal-catalyzed asymmetric reactions. Catalytic, asymmetric cyclopropanation of olefins. J. Am. Chem. Soc..

[8] Dosa P.I., Ruble J.C., Fu G.C. (1997). Planar−Chiral Heterocycles as Ligands in Metal-Catalyzed Processes: Enantioselective Addition of Organozinc Reagents to Aldehydes. J. Org. Chem..

[9] Prier C.K., Rankic D.A., MacMillan D.W.C. (2013). Visible Light Photoredox Catalysis with Transition Metal Complexes: Applications in Organic Synthesis. Chem. Rev..

[10] Tauber J., Imbri D., Opatz T. (2014). Radical Addition to Iminium Ions and Cationic Heterocycles. Molecules.

[11] Minisci F., Bernardi R., Bertini F., Galli R., Perchinummo M. (1971). Nucleophilic character of alkyl radicals—VI: A new convenient selective alkylation of heteroaromatic bases. Tetrahedron.

[12] Minisci F., Mondelli R., Gardini G.P., Porta O. (1972). Nucleophilic character of alkyl radicals—VII. Tetrahedron.

[13] Wang Q.-Q., Xu K., Jiang Y.-Y., Liu Y.-G., Sun B.-G., Zeng C.-C. (2017). Electrocatalytic Minisci Acylation Reaction of *N*-Heteroarenes Mediated by NH4I. Org. Lett..

[14] DiRocco D.A., Dykstra K., Krska S., Vachal P., Conway D.V., Tudge M. (2014). Late-Stage Functionalization of Biologically Active Heterocycles Through Photoredox Catalysis. Angew. Chem. Int. Ed..

[15] Quattrini M.C., Fujii S., Yamada K., Fukuyama T., Ravelli D., Fagnoni M., Ryu I. (2017). Versatile cross-dehydrogenative coupling of heteroaromatics and hydrogen donors via decatungstate photocatalysis. Chem. Commun..

[16] Jin J., MacMillan D.W.C. (2015). Alcohols as alkylating agents in heteroarene C–H functionalization. Nature.

[17] Sherwood T.C., Li N., Yazdani A.N., Dhar T.G.M. (2018). Organocatalyzed, Visible-Light Photoredox-Mediated, One-Pot Minisci Reaction Using Carboxylic Acids via *N*-(Acyloxy)phthalimides. J. Org. Chem..

[18] Cheng W.-M., Shang R., Fu Y. (2017). Photoredox/Brønsted Acid Co-Catalysis Enabling Decarboxylative Coupling of Amino Acid and Peptide Redox-Active Esters with *N*-Heteroarenes. ACS Catal..

[19] Cornella J., Edwards J.T., Qin T., Kawamura S., Wang J., Pan C.-M., Gianatassio R., Schmidt M., Eastgate M.D., Baran P.S. (2016). Practical Ni-Catalyzed Aryl–Alkyl Cross-Coupling of Secondary Redox-Active Esters. J. Am. Chem. Soc..

[20] Huihui K.M.M., Caputo J.A., Melchor Z., Olivares A.M., Spiewak A.M., Johnson K.A., DiBenedetto T.A., Kim S., Ackerman L.K.G., Weix D.J. (2016). Decarboxylative Cross-Electrophile Coupling of *N*-Hydroxyphthalimide Esters with Aryl Iodides. J. Am. Chem. Soc..

[21] Okada K., Okamoto K., Morita N., Okubo K., Oda M. (1991). Photosensitized decarboxylative Michael addition through *N*-(acyloxy)phthalimides via an electron-transfer mechanism. J. Am. Chem. Soc..

[22] Okada K., Okubo K., Morita N., Oda M. (1992). Reductive decarboxylation of *N*-(acyloxy)phthalimides via redox-initiated radical chain mechanism. Tetrahedron Lett..

[23] Uoyama H., Goushi K., Shizu K., Nomura H., Adachi C. (2012). Highly efficient organic light-emitting diodes from delayed fluorescence. Nature.

[24] Singh N., Prasad L.B. (1996). Preparation, Spectroscopic Characterization and Solid State Electrical Conductance of Bimetallic Salts of the Type [M(L—L)3] [M′(MNT)2]. Part II. Synth. React. Inorg. Met.-Org. Chem..

[25] Pratsch G., Lackner G.L., Overman L.E. (2015). Constructing Quaternary Carbons from *N*-(Acyloxy)phthalimide Precursors of Tertiary Radicals Using Visible-Light Photocatalysis. J. Org. Chem..

[26] Scott E., Peter F., Sanders J. (2007). Biomass in the manufacture of industrial products—The use of proteins and amino acids. Appl. Microbiol. Biotechnol..

[27] Williams R. pKa Data Compiled by R. Williams. https://www.chem.wisc.edu/areas/reich/pkatable/pKa_compilation-1-Williams.pdf.

[28] Cheng W.-M., Shang R., Fu M.-C., Fu Y. (2017). Photoredox-Catalysed Decarboxylative Alkylation of *N*-Heteroarenes with *N*-(Acyloxy)phthalimides. Chem. Eur. J..

[29] Isidro-Llobet A., Álvarez M., Albericio F. (2009). Amino Acid-Protecting Groups. Chem. Rev..

[30] Punta C., Minisci F. (2008). Minisci reaction: A Friedel-Crafts type process with opposite reactivity and selectivity. Selective homolytic alkylation, acylation, carboxylation and carbamoylation of heterocyclic aromatic bases. Trends Heterocycl. Chem..

[31] Gottlieb H.E., Kotlyar V., Nudelman A. (1997). NMR Chemical Shifts of Common Laboratory Solvents as Trace Impurities. J. Org. Chem..

[32] Schwarz J., Konig B. (2016). Metal-free, visible-light-mediated, decarboxylative alkylation of biomass-derived compounds. Green Chem..

[33] Garrido-Castro A.F., Choubane H., Daaou M., Maestro M.C., Aleman J. (2017). Asymmetric radical alkylation of *N*-sulfinimines under visible light photocatalytic conditions. Chem. Commun..

[34] Hassall C.H. (1948). 13. The action of hydrogen peroxide on symmetrical triketones. J. Chem. Soc..

[35] Guo Z., Jiang X., Jin C., Zhou J., Sun B., Su W. (2017). Copper-Catalyzed Highly Efficient Esterification of Aldehydes with *N*-Hydroxyphthalimide via Cross-Dehydrogenative Coupling in Water at Room Temperature. Synlett.

[36] Fang L., Chen L., Yu J., Wang L. (2015). Benzoyl Peroxide Promoted Radical ortho-Alkylation of Nitrogen Heteroaromatics with Simple Alkanes and Alcohols. Eur. J. Org. Chem..

[37] Tang R.-J., Kang L., Yang L. (2015). Metal-Free Oxidative Decarbonylative Coupling of Aliphatic Aldehydes with Azaarenes: Successful Minisci-Type Alkylation of Various Heterocycles. Adv. Synth. Catal..

[38] Paul S., Guin J. (2015). Dioxygen-Mediated Decarbonylative C–H Alkylation of Heteroaromatic Bases with Aldehydes. Chem. Eur. J..

[39] Yu C., Lee K., You Y., Cho E.J. (2013). Synthesis of 2-Substituted Benzothiazoles by Visible Light-Driven Photoredox Catalysis. Adv. Synth. Catal..

[40] Babu K.R., Zhu N., Bao H. (2017). Iron-Catalyzed C–H Alkylation of Heterocyclic C–H Bonds. Org. Lett..

