# Supplementary material for: A Visible Light-Driven Minisci-Type Reaction with N-Hydroxyphthalimide Esters
Source: Molecules. 2018 Mar 27;23(4):764. doi: 10.3390/molecules23040764 (PMC6017455; doi:10.3390/molecules23040764)
Supplement: Supplementary file 1 [file molecules-23-00764-s001.pdf]

A Visible Light-Driven Minisci-Type Reaction with N-Hydroxyphthalimide Esters

Lisa Marie Kammer, Aliyaah Rahman and Till Opatz*

Institute of Organic Chemistry, Johannes Gutenberg University,
Duesbergweg 10-14, 55128 Mainz, Germany

Supporting Information

Table of Contents

I. Reaction Setup for Irradiations S2

II. Optimization Studies and Control Experiments S3

III. UV-Vis Spectroscopy Data S5

IV. NMR Spectra S10

I. Reaction Setup for Irradiations and Emission spectra

Irradiation was carried out using a blue LED (high power 100 W LED, HPR40E-48K100BG (GalnN/GaN) from Huey Jann Electronic Industry CO,LTD., Taiwan, λ_{max} = 462 ± 3 nm). The lamp was placed in a distance of 5 cm from the reaction vessel. No further cooling was necessary to maintain ambient temperature. The setup and the emission spectra are shown in the following pictures.

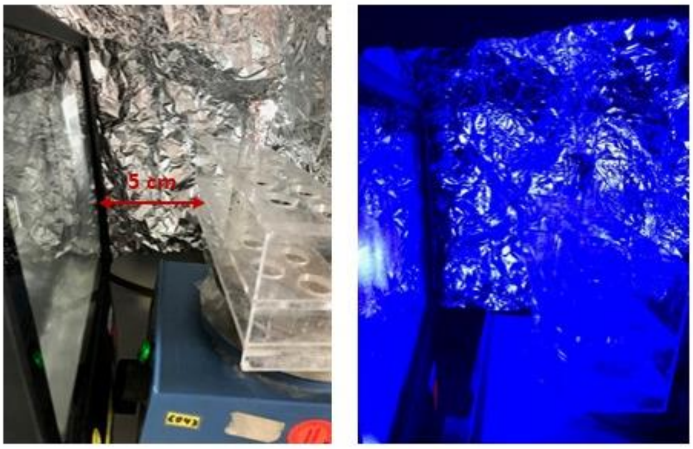

Figure S1. Setup for irradiation with a 100 W blue LED module

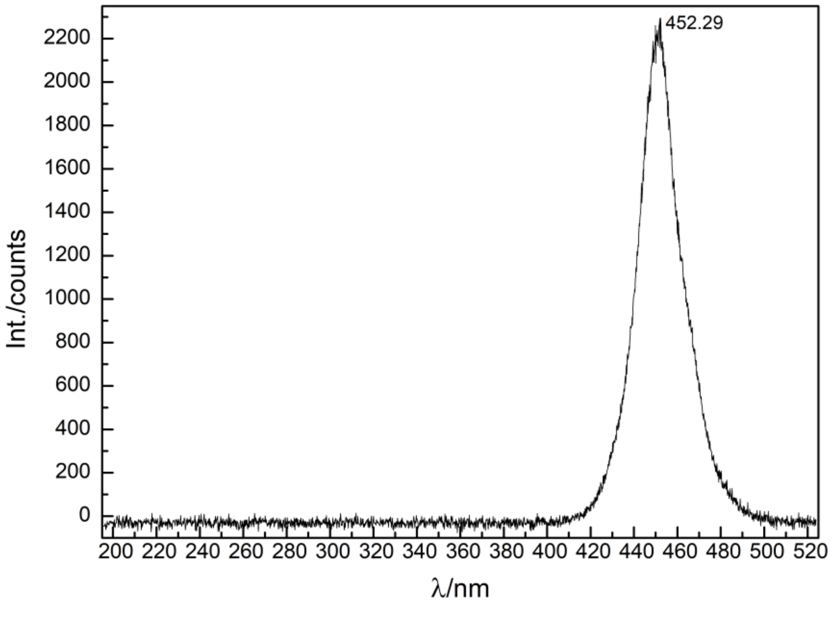

Figure S2. Emission spectra of the 100 W blue LED module. The spectra was recorded with an USB2000+ from Ocean Optics, Inc., Dunedin, Florida, USA. Wavelength range from 200–523 nm, optical resolution 1 nm.

II. Optimization Studies and Control Experiments

Table S1. Catalyst loading study

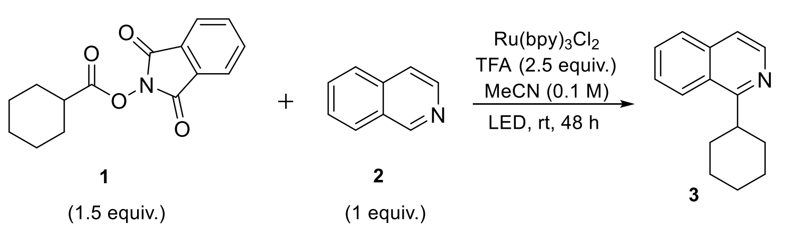

entry	catalyst loading (mol%)	yield (%)[a]
1	2.00	24%
**2**	**1.00**	**27%**
3	0.50	7%
4	0.25	6%
5	0.025	traces[b]

Procedure: The reactions were performed in a glass vial according to the General Procedure B using *N*-(cyclohexylcarbonyloxy)phthalimide (**1**, 82.0 mg, 0.3 mmol), isoquinoline (**2**, 25.8 mg, 0.2 mmol), $Ru(bpy)_3Cl_2$, TFA (0.05 mL, 0.6 mmol) and acetonitrile (3 mL). [a] Isolated yield. [b] Judged by HPLC.

Table S2. Solvent screening for the photoredox Minisci-type reaction

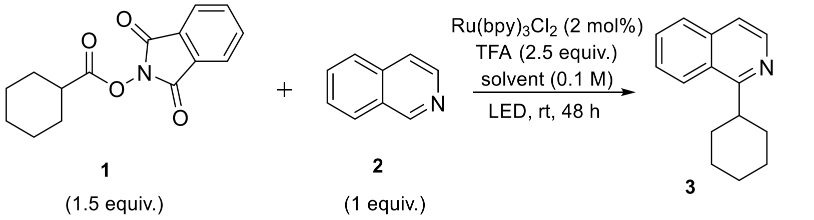

entry	solvent	yield (%)[a]
1	DCM	/
2	MeCN	24%
3	MeCN/H_2O (1:1)	36%
4	MeOH	43%
5	DMA	79%
6	DMSO	80%
**7**	**DMF**	**91%**

Procedure: The reactions were performed in a glass vial according to the General Procedure B using *N*-(cyclohexylcarbonyloxy)phthalimide (**1**, 82.0 mg, 0.3 mmol), isoquinoline (**2**, 25.8 mg, 0.2 mmol), $Ru(bpy)_3Cl_2$ (4 mg, 2 mol%), TFA (0.05 mL, 0.6 mmol) and the indicated dry solvent (3 mL). [a] Isolated yield.

Table S3. Additive screening for the photoredox Minisci-type reaction

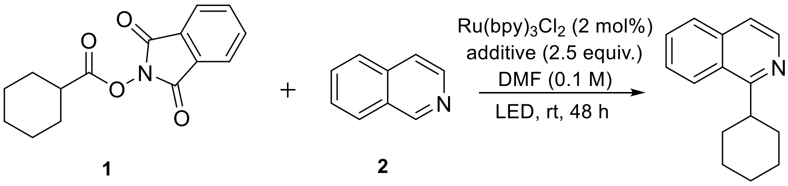

1 (1.5 equiv.) **2** (1 equiv.) **3**

entry	additive	yield (%)[a]
1	HOAc	/
2	HOAc[b]	/
3	[c]	31%
4	H_2SO_4	70%
5	HCl	71%
6	AlCl3	74%
7	$In(OTf)_3$	79%
8	TFA	91%
**9**	***p*-TsOH monohydrate**[d]	**quant.**

Procedure: The reactions were performed as described for table S1. [a] Isolated yield. [b] The reaction was performed with 1.5 equiv. of acetic acid and without any further solvent. [c] isoquinolinium triflate (1 equiv.) was used instead of **2**. [d] Same yield was achieved using 2.50 and 1.50 equiv. of the additive. With 1.0 equiv. the yield decreased to 72%.

Table S4. Equivalent screening of isoquinoline

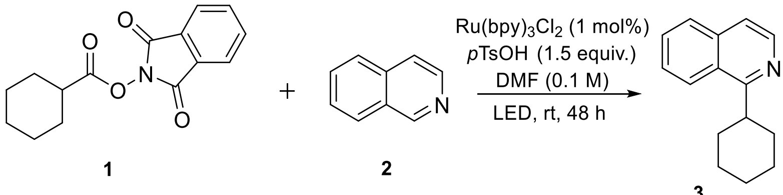

1 (1.5 equiv.) **2** (**X** equiv.) **3**

entry	equiv. **2**	yield (%)[a]
1	2.00	93%
2	1.50	95%
**3**	**1.00**	**quant**

Procedure: The reactions were performed as described for table S1. [a] Isolated yield.

III. UV-Vis Spectroscopy

Absorption spectra of *N*-(cyclohexylcarbonyloxy)phthalimide, isoquinoline and *p*-toluenesulfonic acid monohydrate, as well as the spectra of the reaction mixture before irradiation are depicted in figure S3.

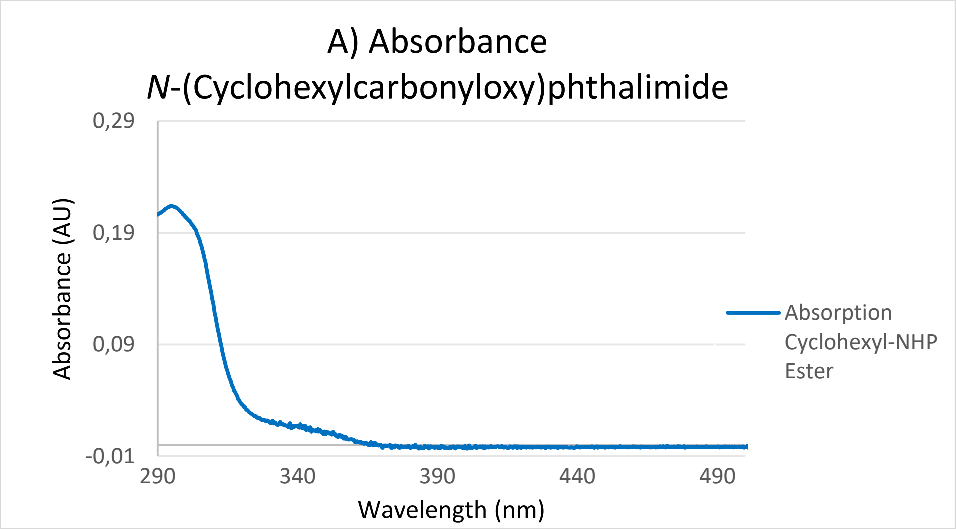

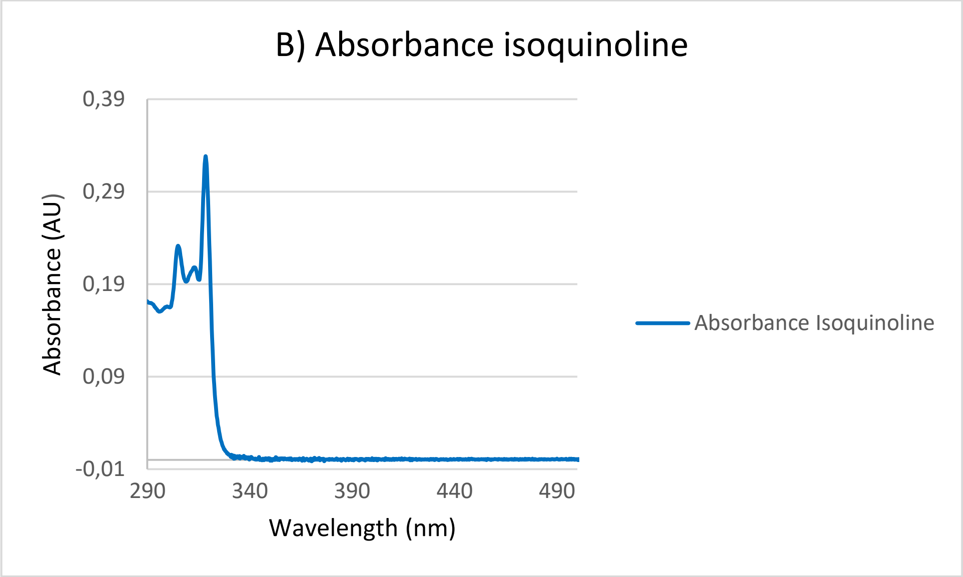

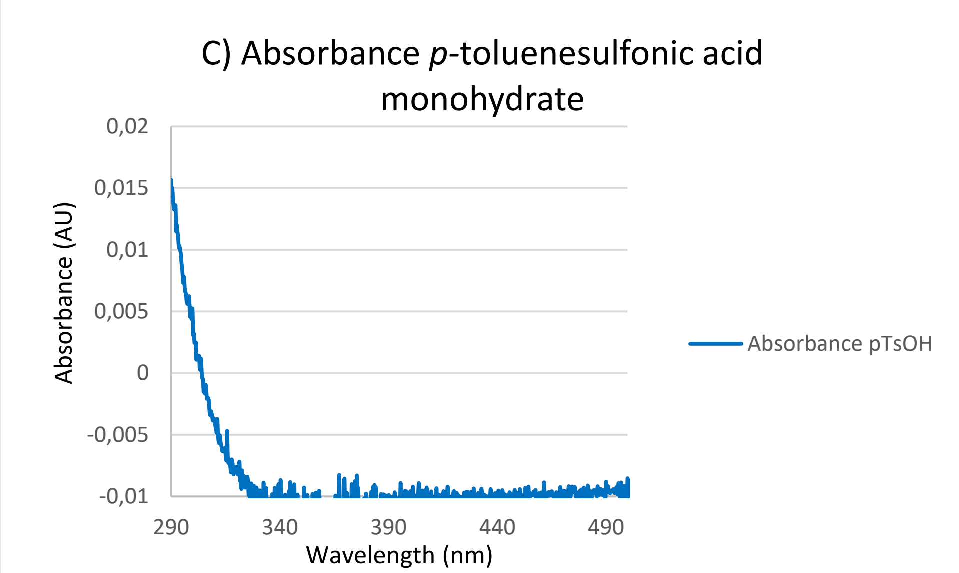
C) Absorbance *p*-toluenesulfonic acid monohydrate
Absorbance (AU)
0,02
0,015
0,01
0,005
0
-0,005
-0,01
290
340
390
440
490
Wavelength (nm)
Absorbance pTsOH

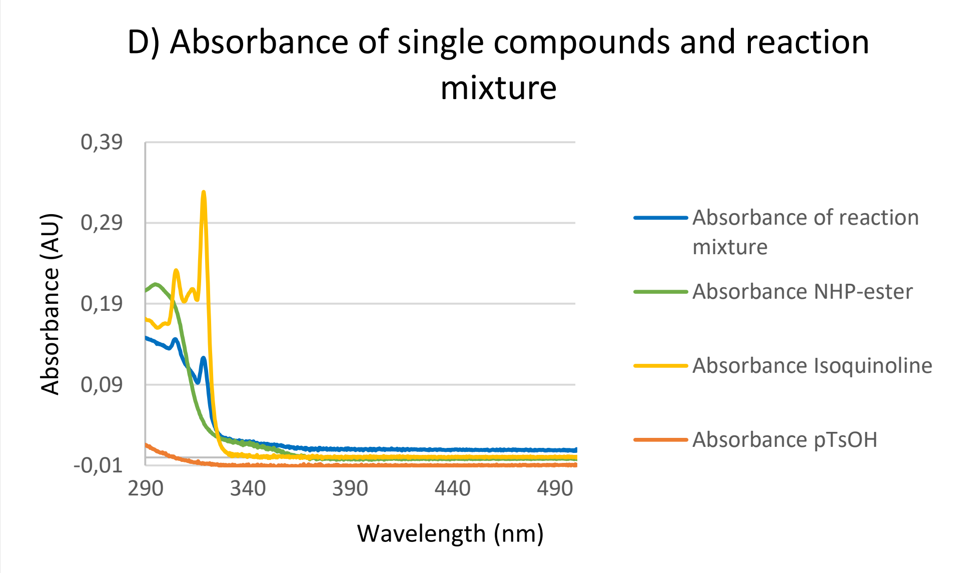
D) Absorbance of single compounds and reaction mixture
Absorbance (AU)
0,39
0,29
0,19
0,09
-0,01
290
340
390
440
490
Wavelength (nm)
Absorbance of reaction mixture
Absorbance NHP-ester
Absorbance Isoquinoline
Absorbance pTsOH

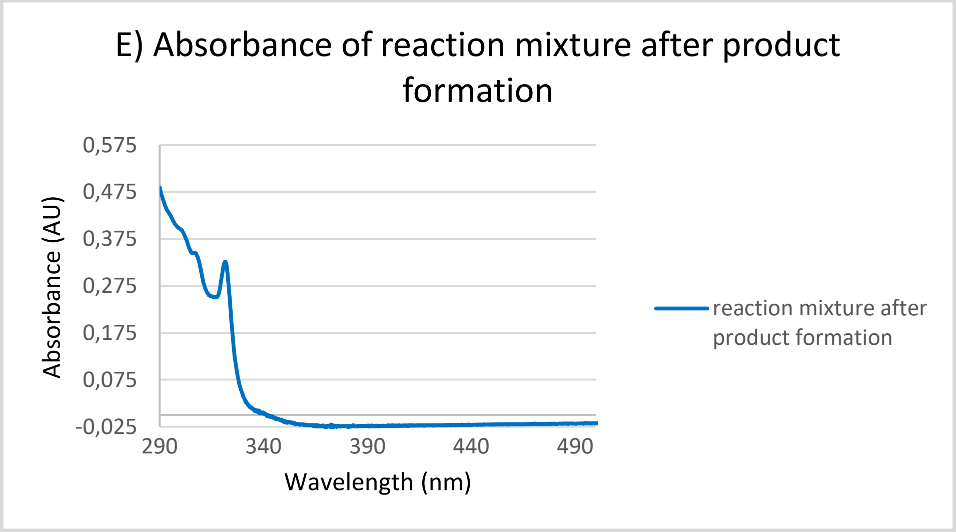

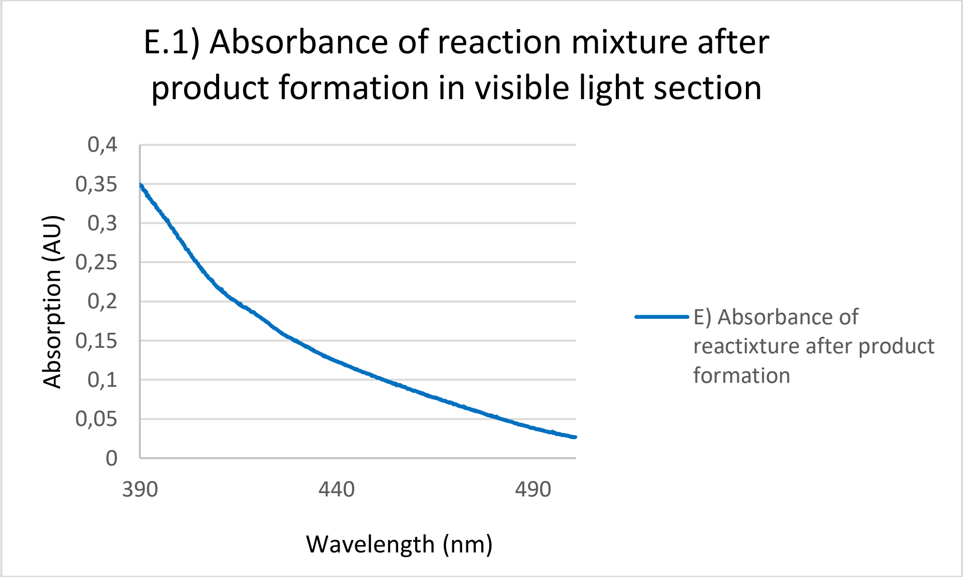

Figure S3. UV-Vis absorption spectra at 290–500 nm measured for A) *N*-(cyclohexylcarbonyloxy)phthalimid ($1 \cdot 10^{-4}$ M), B) isoquinoline ($1 \cdot 10^{-4}$ M), *p*-toluenesulfonic acid monohydrate ($1 \cdot 10^{-4}$ M), D) the reaction mixture ($1 \cdot 10^{-4}$ M), E) reaction mixtures after product formation (yellowish solution, $1 \cdot 10^{-4}$ M) and E.1) UV-Vis absorption spectra at 390–500 nm of the reaction mixture after product formation (0.1 M). All spectra were measured in dry DMF.

The obtained spectroscopic data shows that the absorption maxima of each compound lie well below 350 nm (figure S3). In all cases, the absorption of each component and mixtures was measured using the same stoichiometric ratio as applied in the general procedure B. Upon product formation the color of the reaction mixture changed from colorless to yellow and further UV-Vis spectra were recorded (figure S3, E and E.1). The overlapping absorption maxima of the single compounds in the area between 290–350 nm did not change, but a new absorption was detected stretching out into the visible range. The occurrence of potential ground state charge-transfer-complexes, which may act as photocatalysts, was investigated by comparing the absorption spectra of mixtures of the reactants with the absorption spectra of the pure compounds (figure S4).

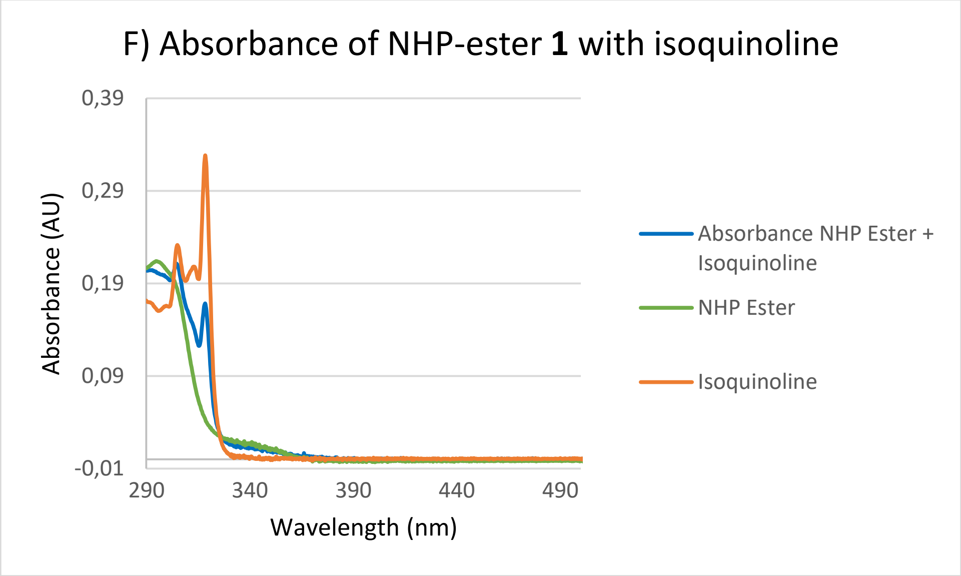
F) Absorbance of NHP-ester 1 with isoquinoline
Absorbance (AU)
0,39
0,29
0,19
0,09
-0,01
290
340
390
440
490
Wavelength (nm)
Absorbance NHP Ester + Isoquinoline
NHP Ester
Isoquinoline

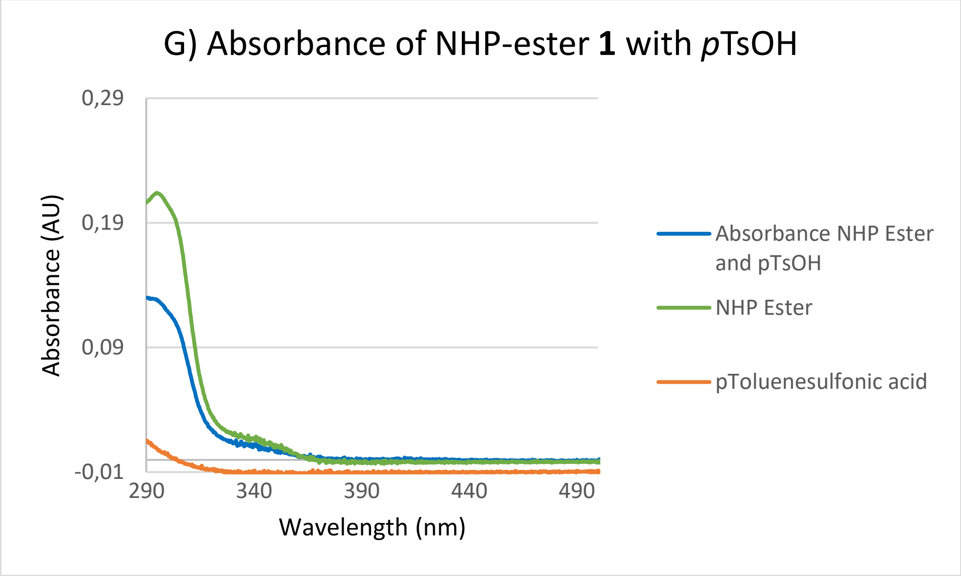
G) Absorbance of NHP-ester 1 with pTsOH
Absorbance (AU)
0,29
0,19
0,09
-0,01
290
340
390
440
490
Wavelength (nm)
Absorbance NHP Ester and pTsOH
NHP Ester
pToluenesulfonic acid

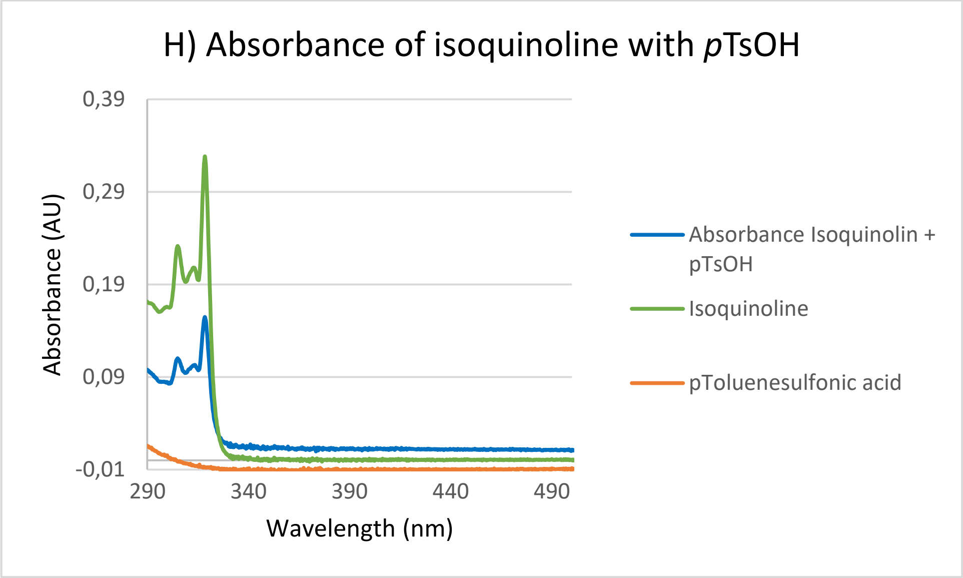

Figure S4. Exclusion of ground state charge-transfer complexes. UV-Vis absorption spectra at 290–500 nm measured for different mixtures of *N*-(cyclohexylcarbonyloxy)phthalimid ($1 \cdot 10^{-4}$ M), B) isoquinoline ($1 \cdot 10^{-4}$ M), *p*-toluenesulfonic acid monohydrate ($1 \cdot 10^{-4}$ M) and D) the reaction mixture ($1 \cdot 10^{-4}$ M). All spectra were measured in dry. DMF.

The spectra of the two component mixtures (blue) don´t show any new absorption maxima in comparison to the spectras measured for the single compounds (green, orange).

IV. NMR Spectra

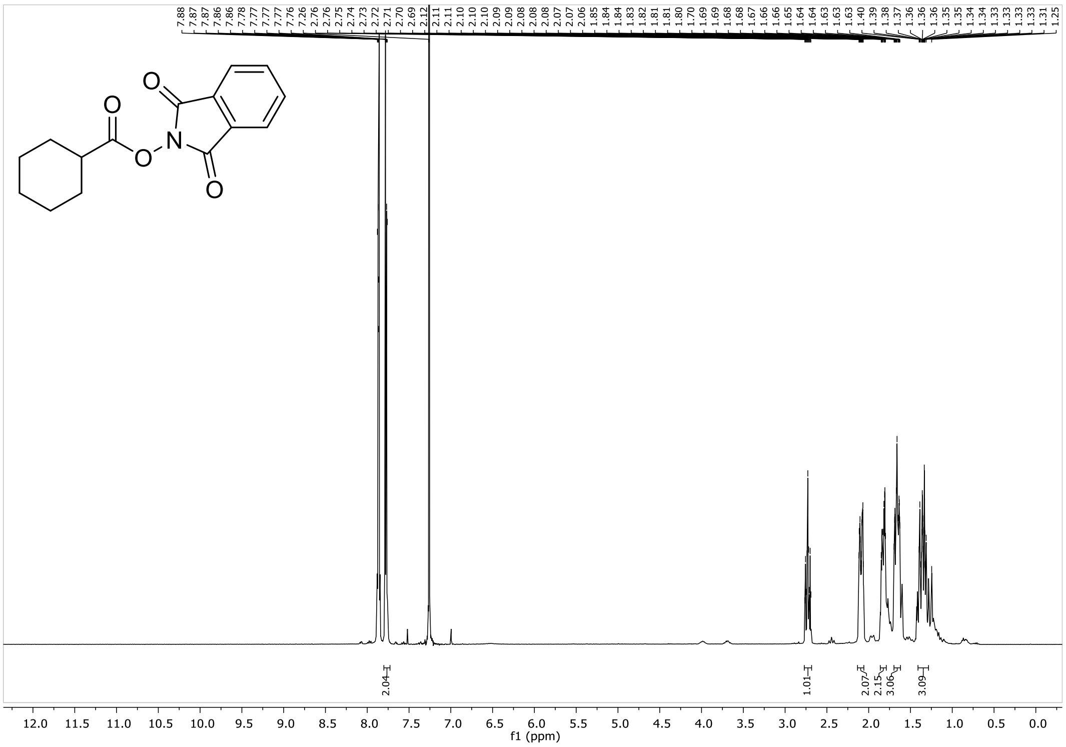

Figure S5. ^{1}H-NMR in $CDCl_3$ *N*-(Cyclohexyl-2-carbonyloxy)phthalimide (**1**)

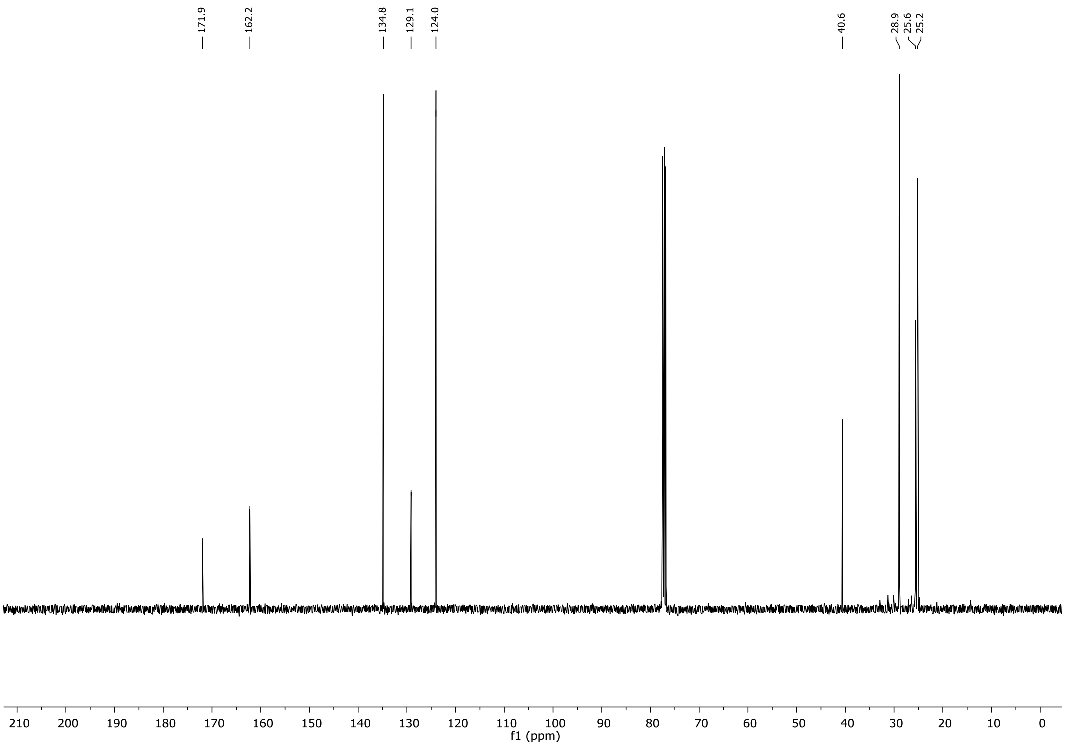

Figure S6. ^{13}C-NMR in $CDCl_3$ *N*-(Cyclohexyl-2-carbonyloxy)phthalimide (**1**)

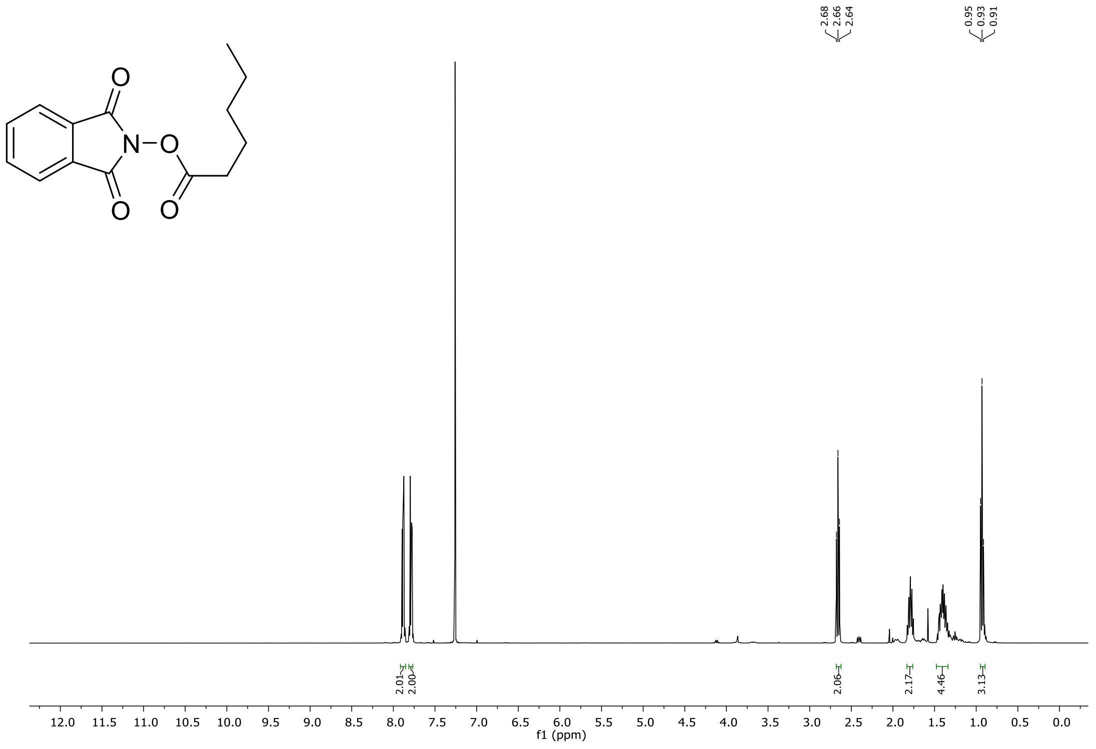

Figure S7. ^{1}H-NMR in $CDCl_3$ *N*-(Hexanoyloxy)phthalimide (**26**)

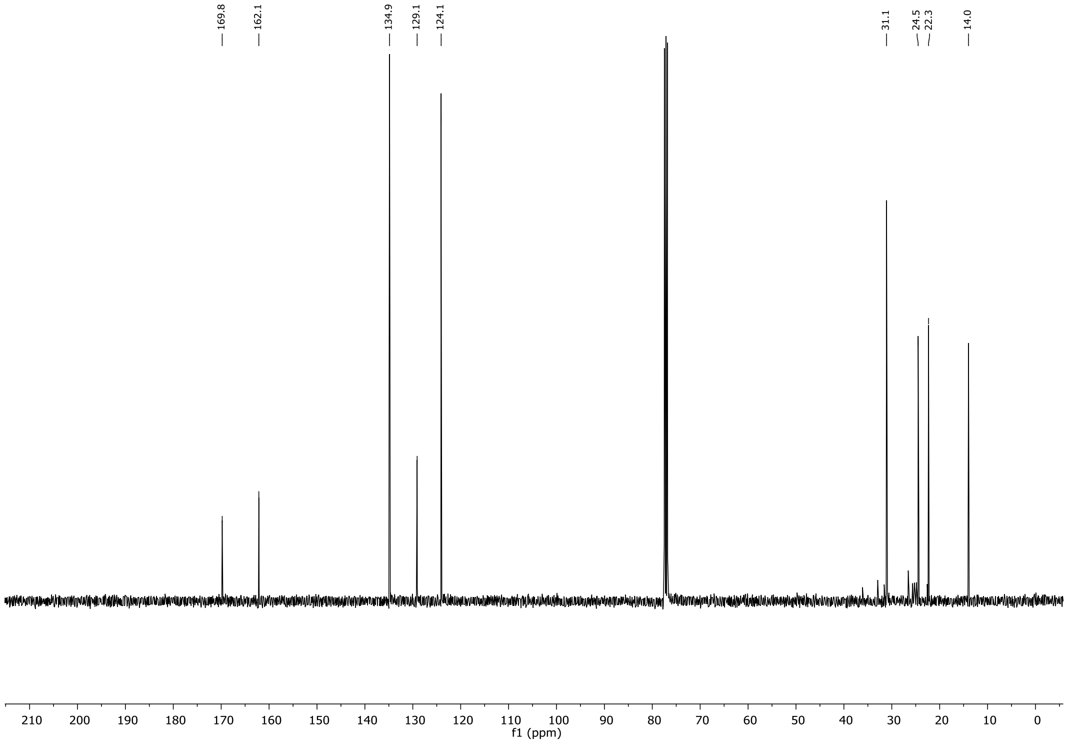

Figure 8. ^{13}C-NMR in $CDCl_3$ *N*-(Hexanoyloxy)phthalimide (**26**)

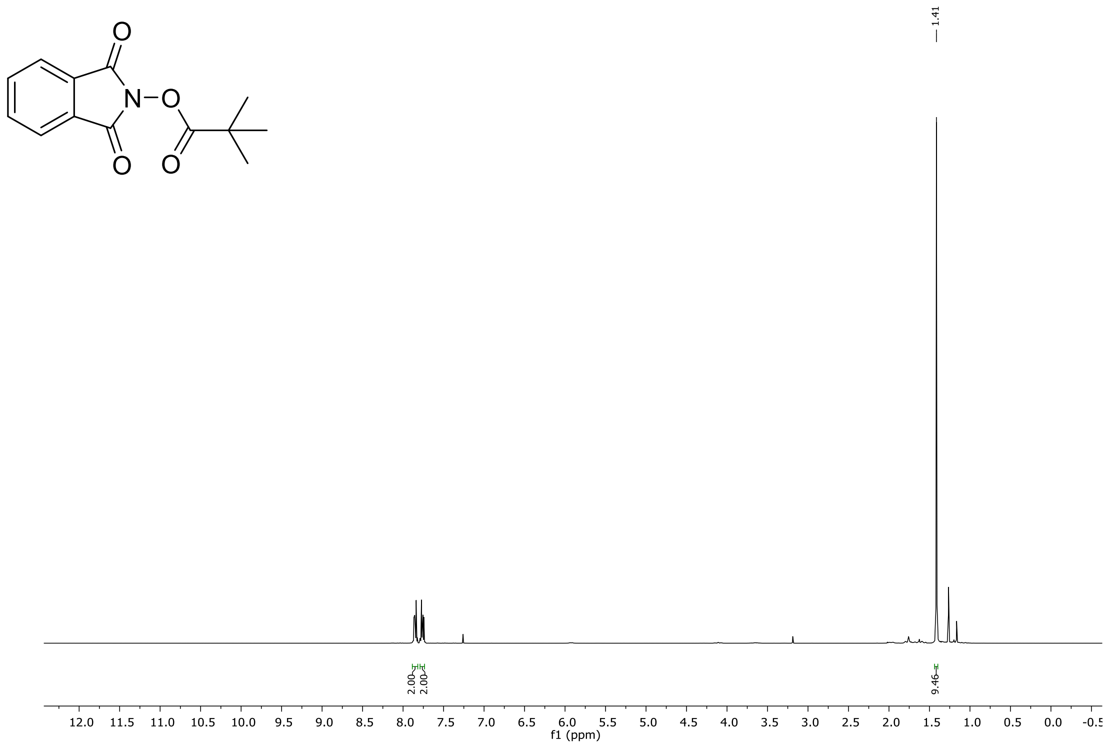

Figure S9. ^{1}H-NMR in $CDCl_3$ *N*-(Pivaloyloxy)phthalimide (**27**)

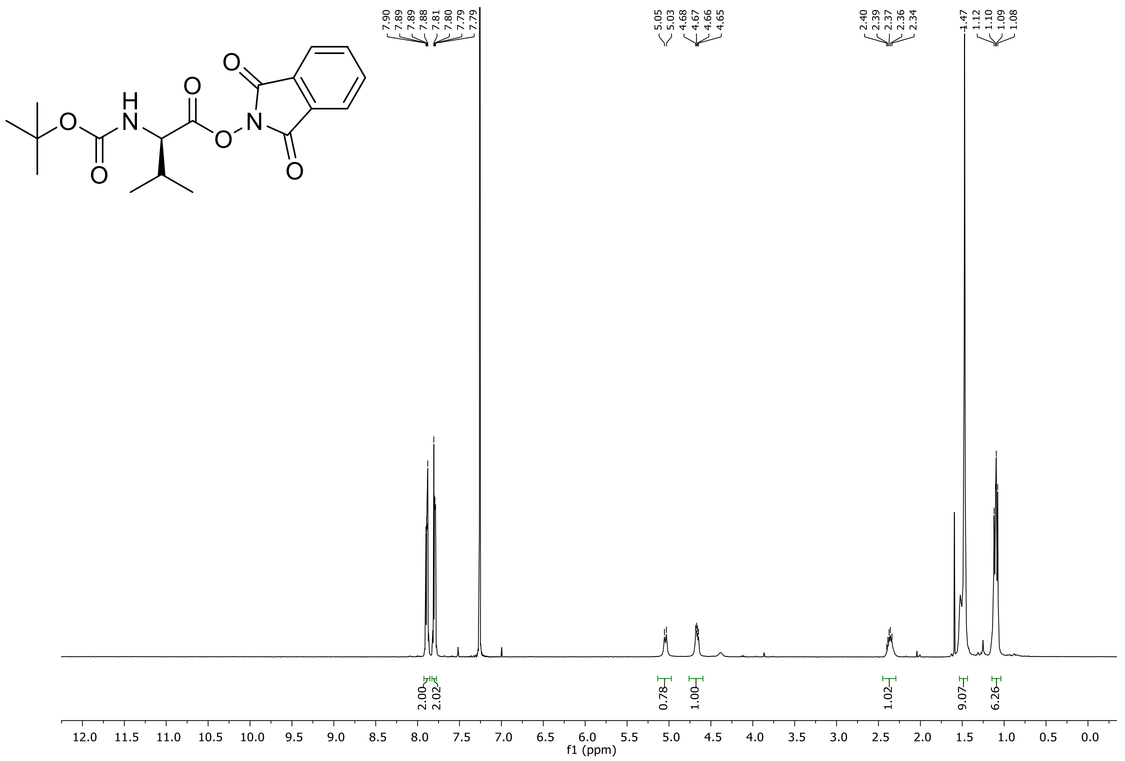

Figure S10. ^{1}H-NMR in $CDCl_3$ *N*-(*N-tert*-Butoxycarbonyl-L-valinyloxy)phthalimide (**28**)

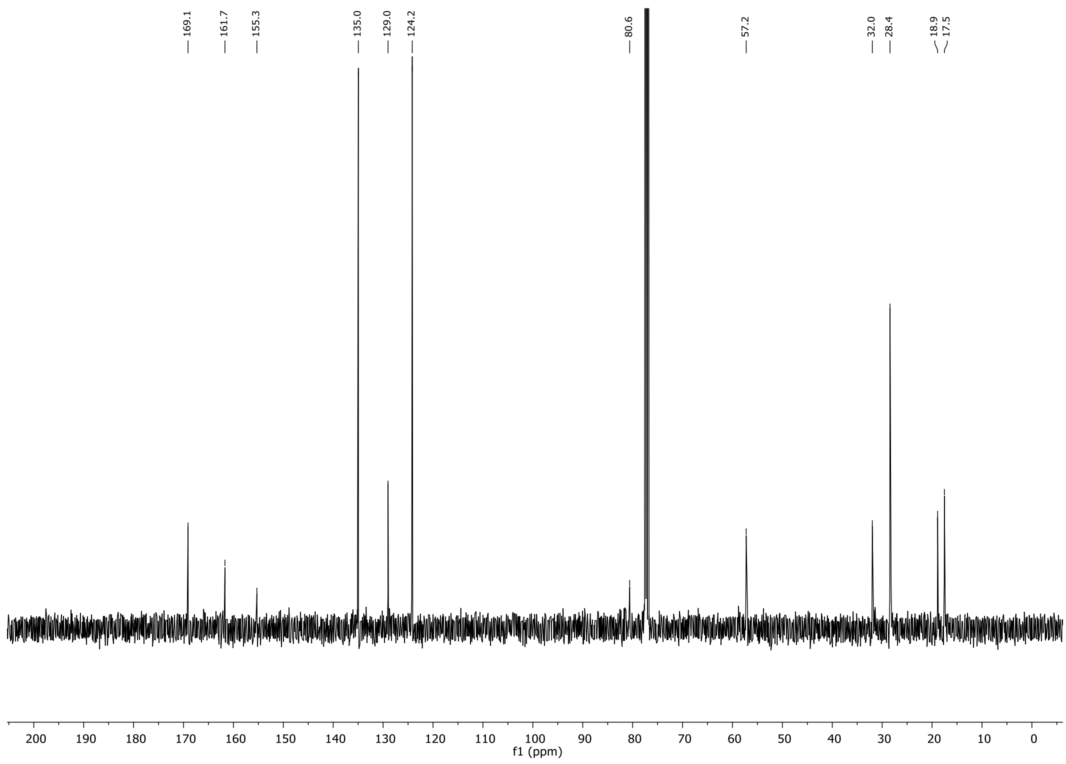

Figure S11. ^{13}C-NMR in $CDCl_3$ *N*-(*N*-*tert*-Butoxycarbonyl-L-valinyloxy)phthalimide (**28**)

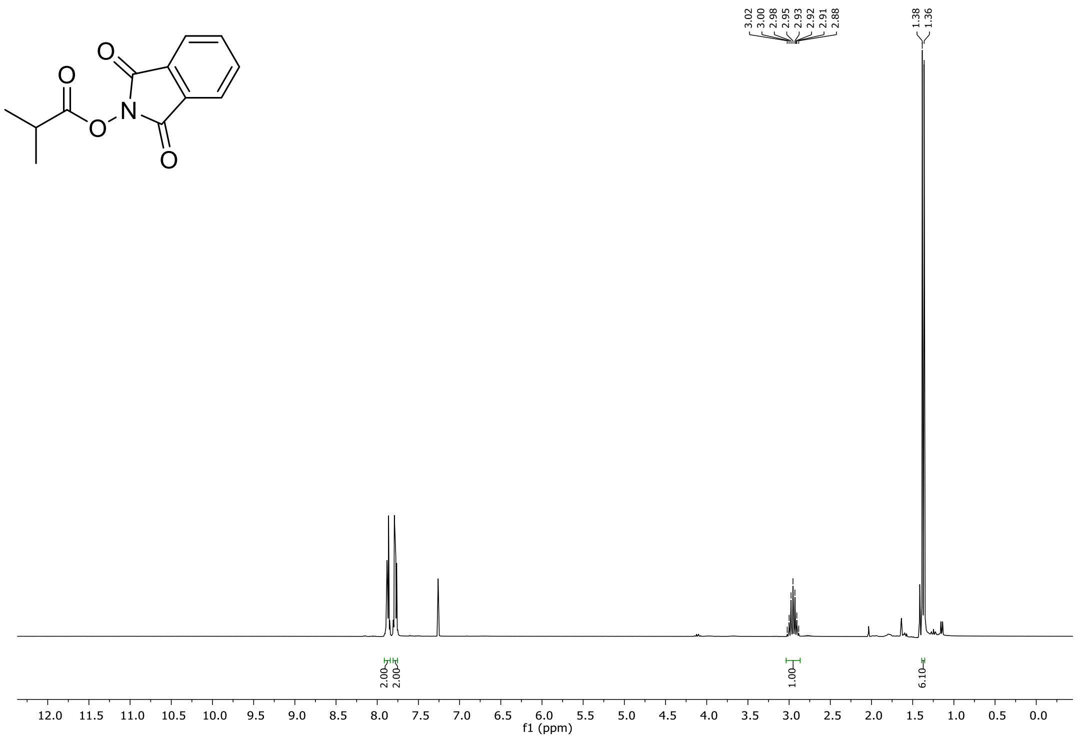

Figure S12. ^{1}H-NMR in $CDCl_3$ *N*-(Isobutyryloxy)phthalimide (**29**)

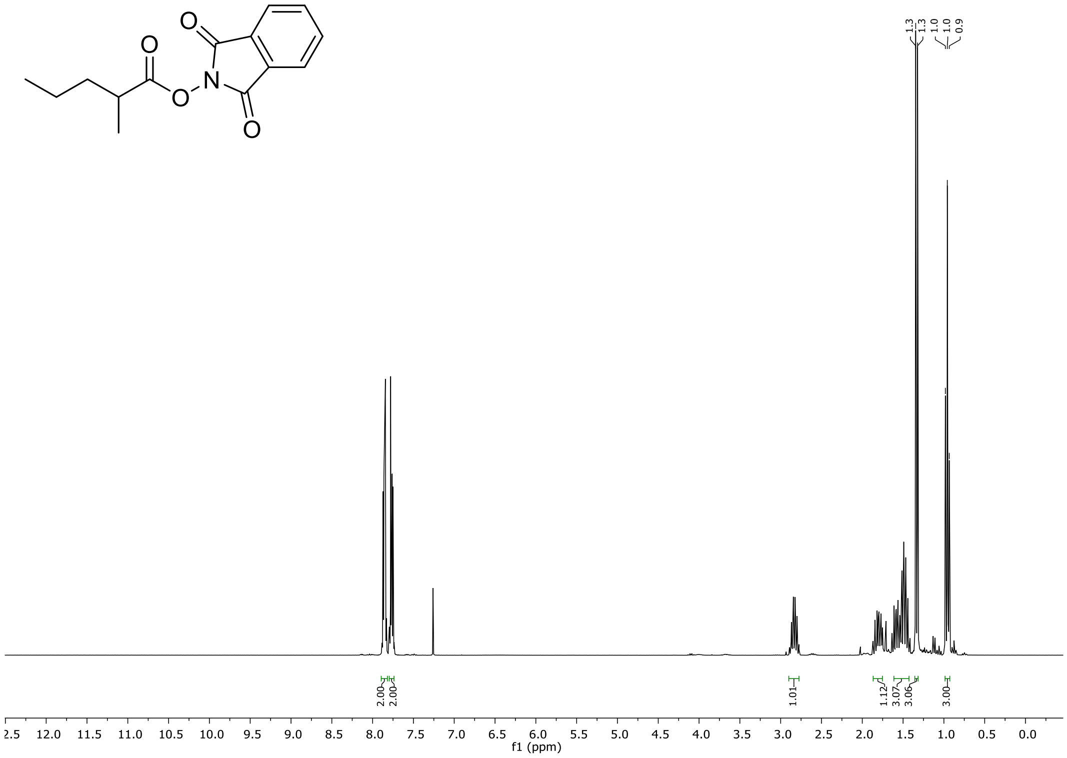

Figure S13. ^{1}H-NMR in $CDCl_3$ *N*-(Pentyl-2-carbonyloxy)phthalimide (**30**)

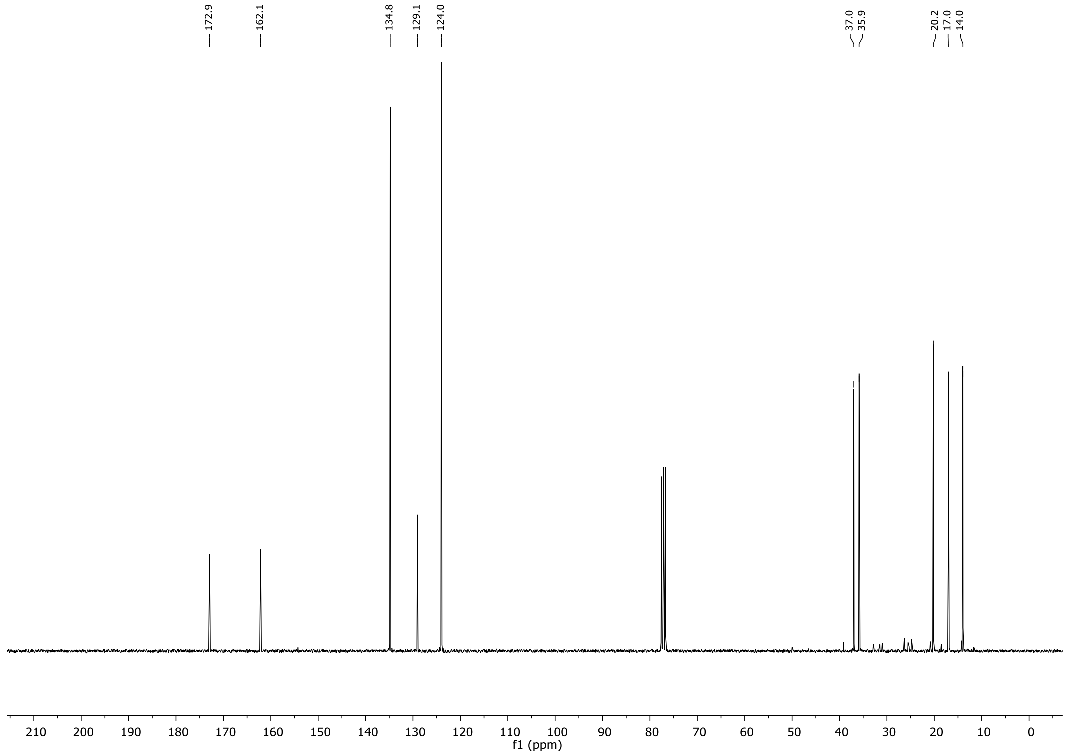

Figure S14. ^{13}C-NMR in $CDCl_3$ *N*-(Pentyl-2-carbonyloxy)phthalimide (**30**)

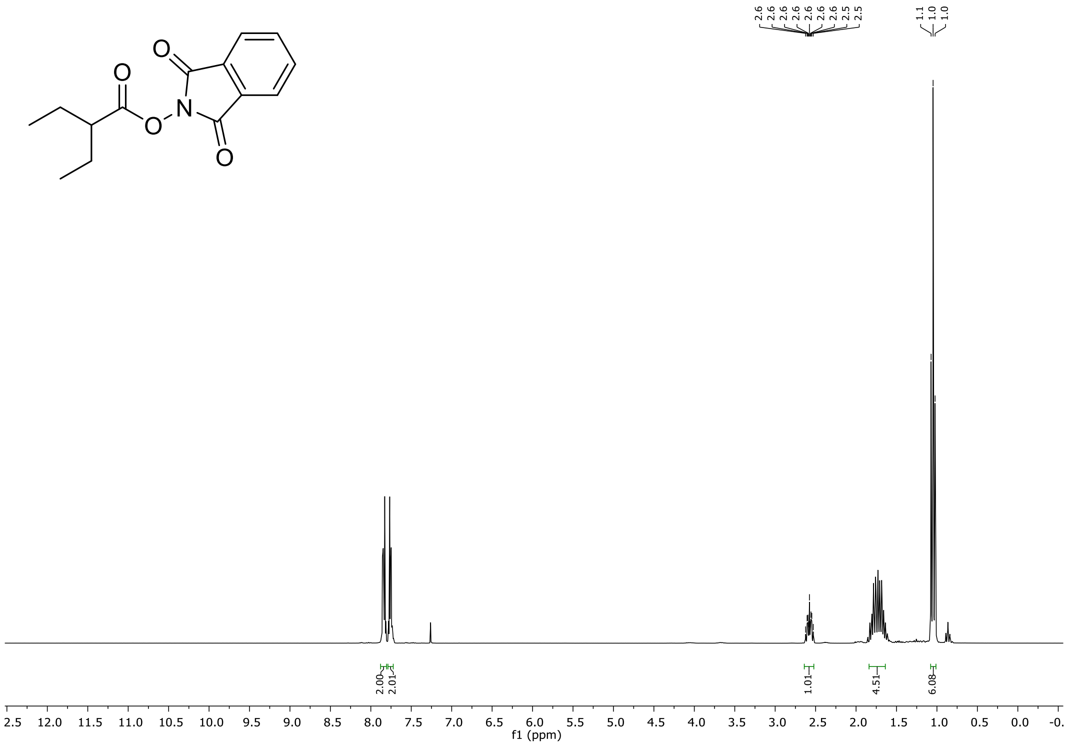

Figure S15. ^{1}H-NMR in $CDCl_3$ *N*-(2-Ethylbutyryloxy)phthalimide (**31**)

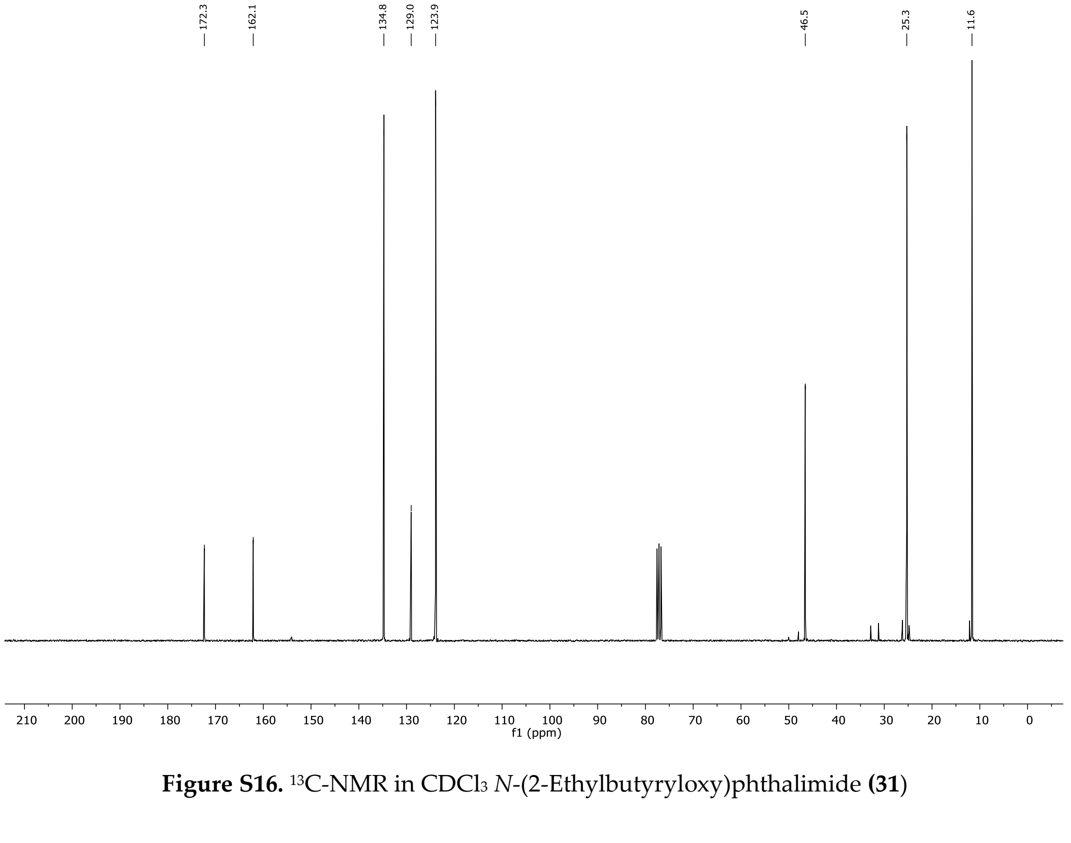

Figure S16. ^{13}C-NMR in $CDCl_3$ *N*-(2-Ethylbutyryloxy)phthalimide **(31**)

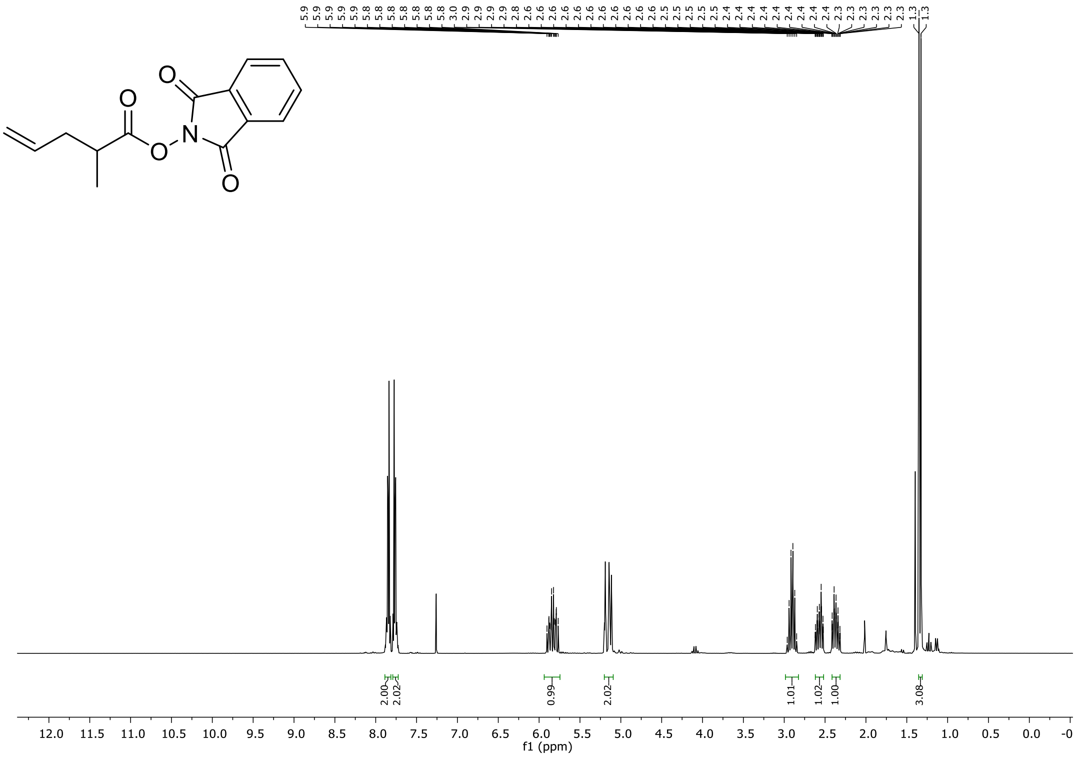

Figure S17. ^{1}H-NMR in $CDCl_3$ *N*-(Pent-4-enyl-2-carbonyloxy)phthalimide (**32**)

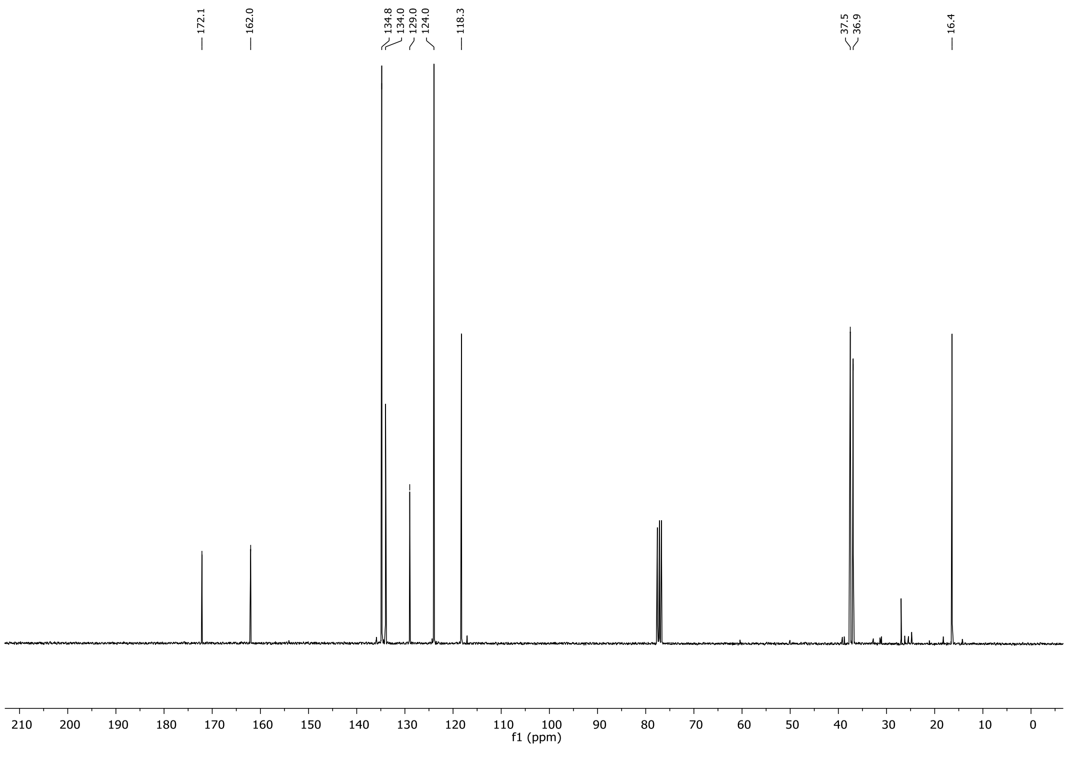

Figure S18. ^{13}C-NMR in $CDCl_3$ *N*-(Pent-4-enyl-2-carbonyloxy)phthalimide (**32**)

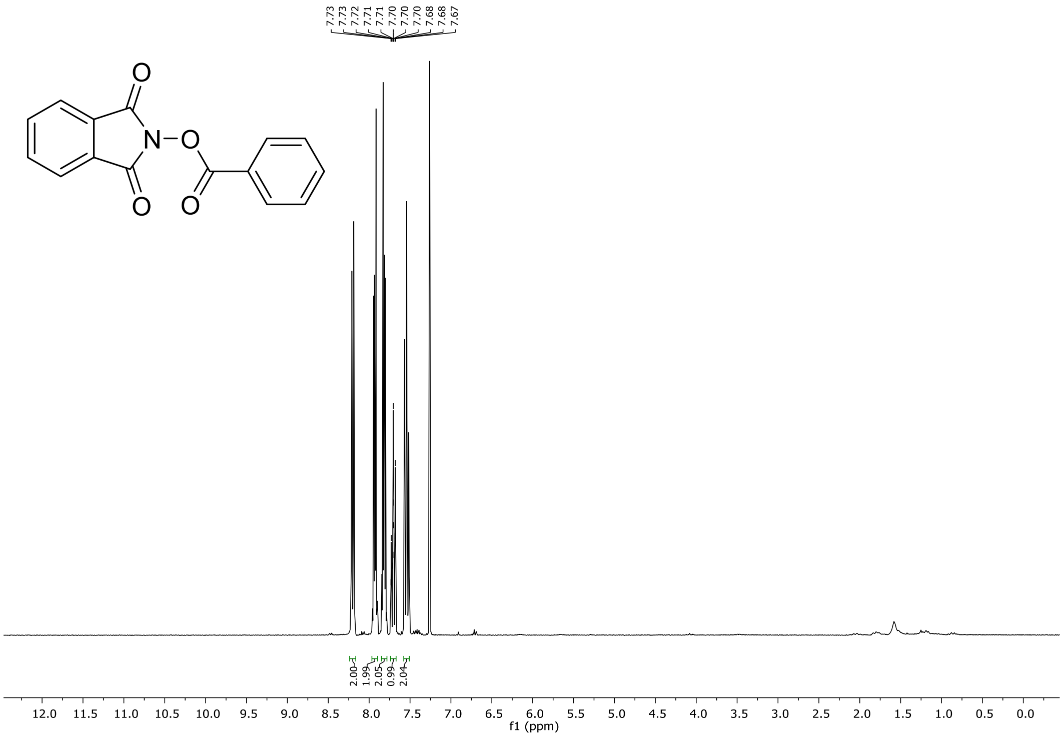

Figure S19. ¹H-NMR in $CDCl_3$ *N*-(Benzoyloxy)phthalimide (**33**)

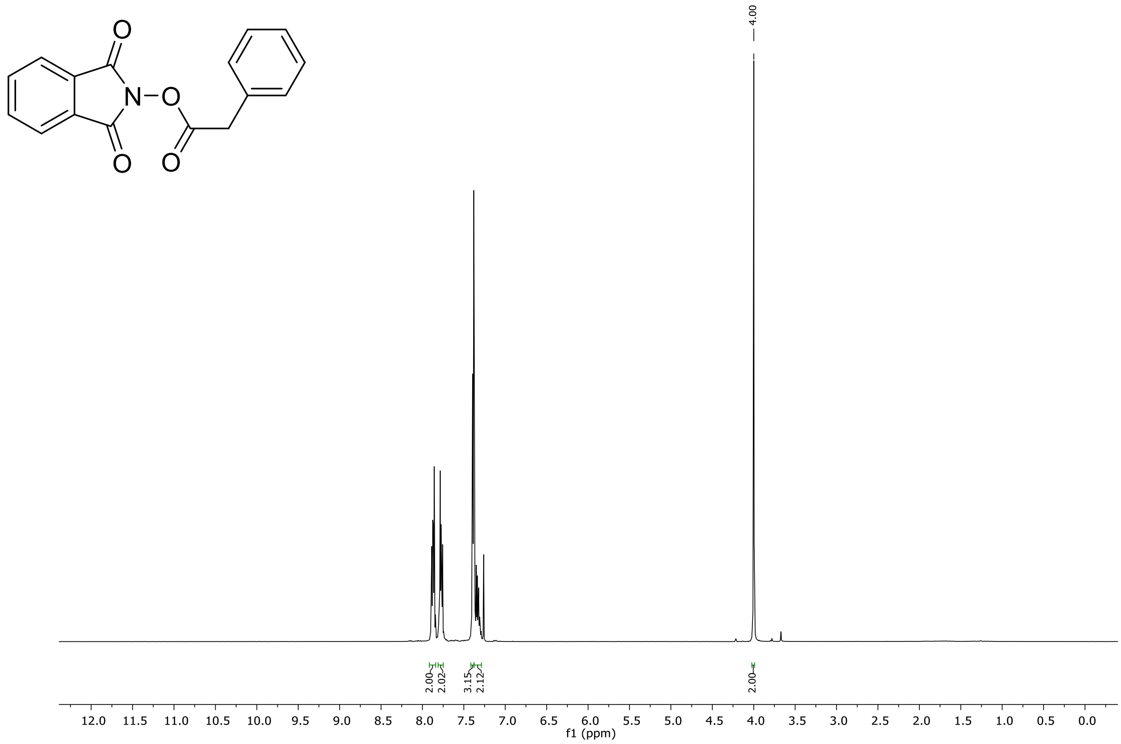

Figure S20. ¹H-NMR in $CDCl_3$ *N*-(2-Phenylacetyloxy)phthalimide (**34**)

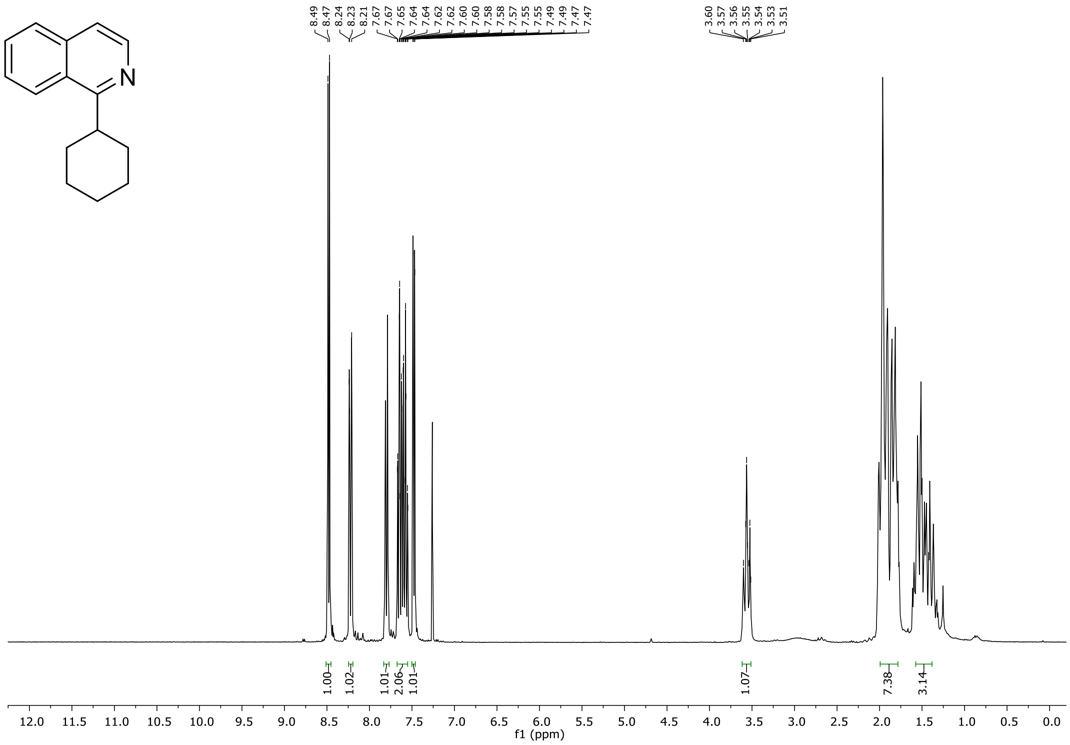

Figure S21. ^{1}H-NMR in $CDCl_3$ 1-(Cyclohexyl)isoquinoline (**3**)

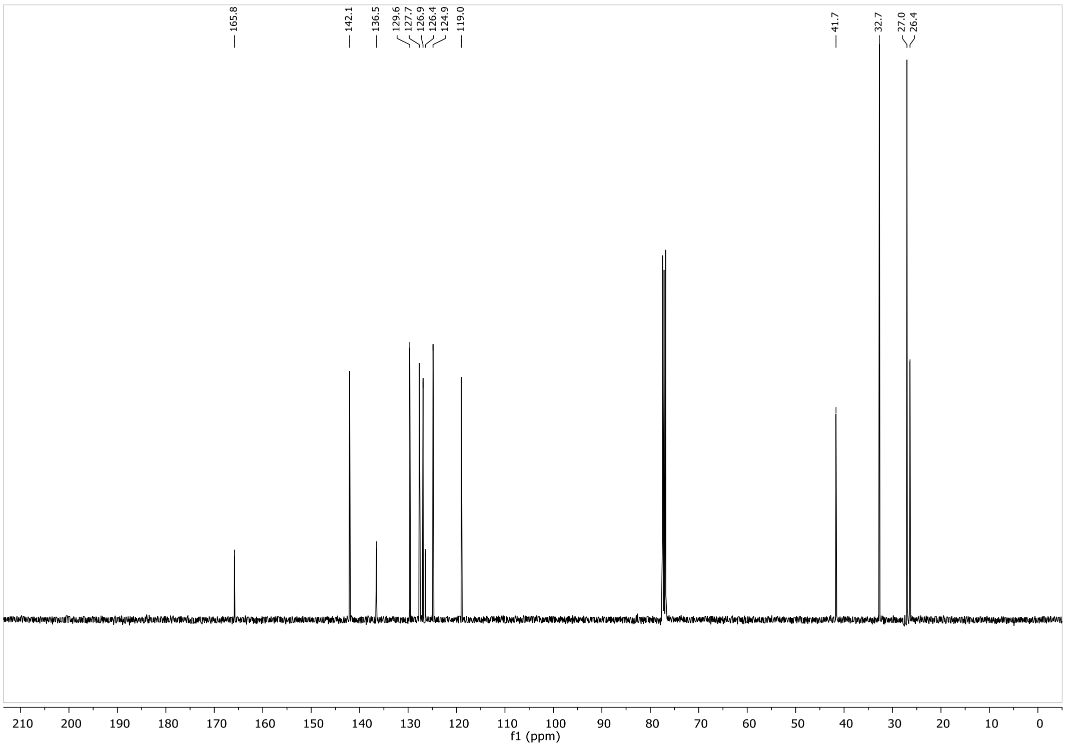

Figure S22. ^{13}C-NMR in $CDCl_3$ 1-(Cyclohexyl)isoquinoline (**3**)

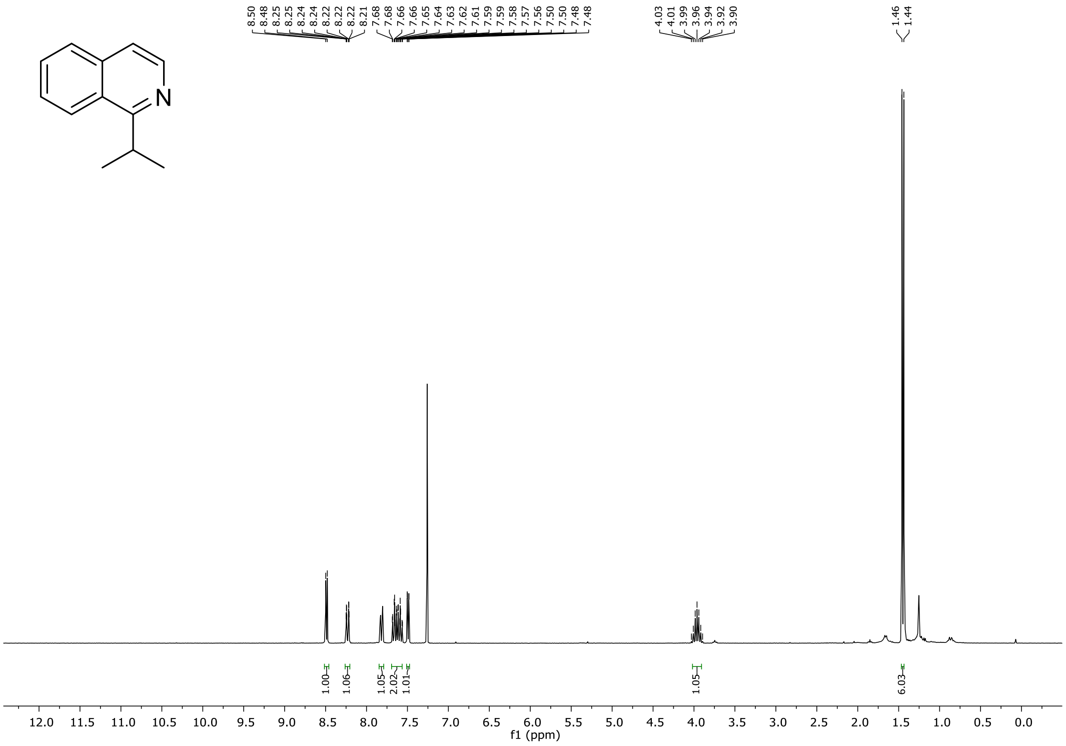

Figure S23. ^{1}H-NMR in $CDCl_3$ 1-(Propan-2-yl)isoquinoline (**5**)

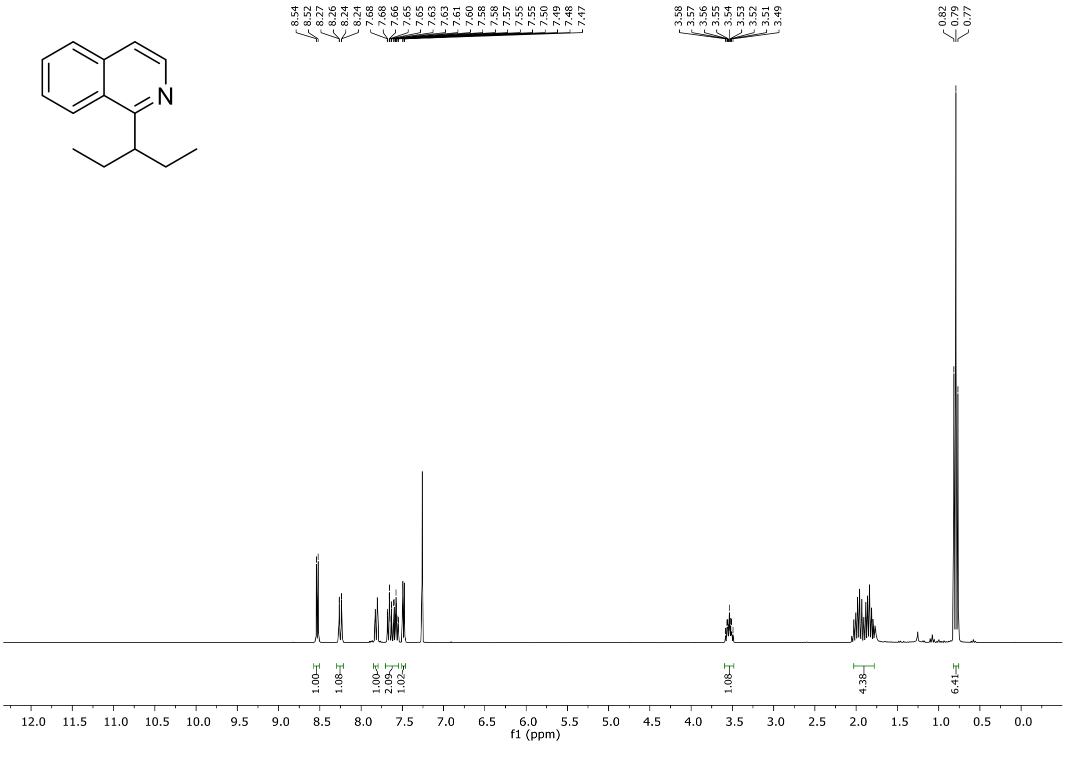

Figure S24. ^{1}H-NMR in $CDCl_3$ 1-(Pentan-3-yl)isoquinoline (**6**)

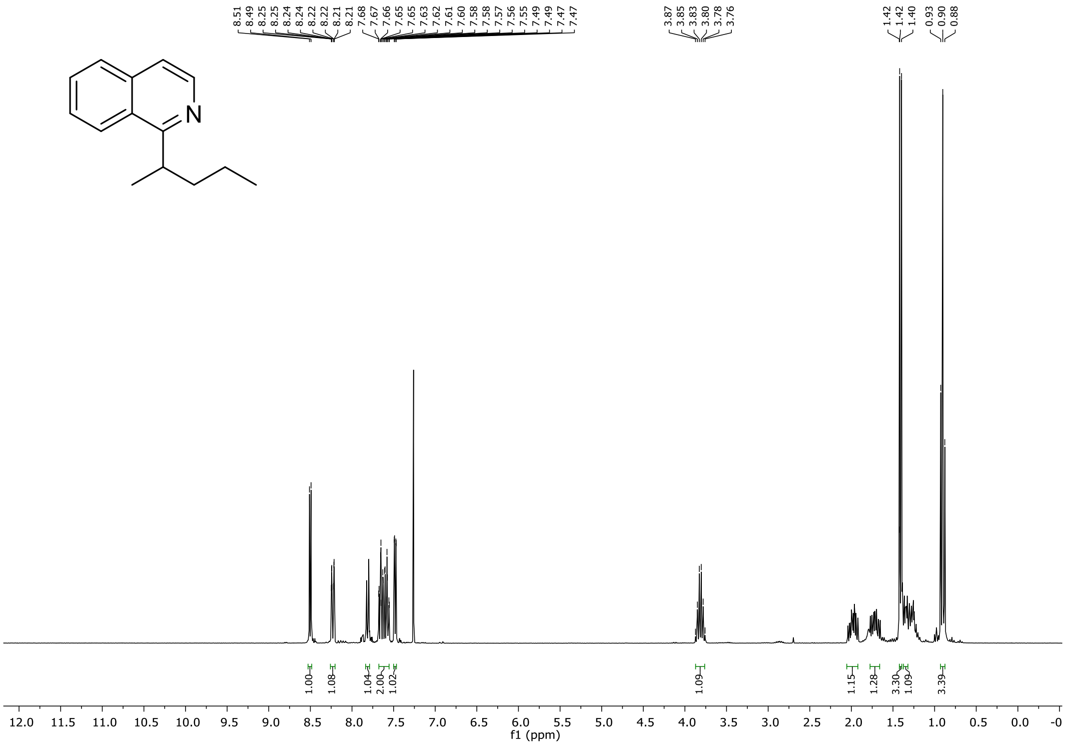

Figure S25. ^{1}H-NMR in $CDCl_3$ 1-(Pentan-2-yl)isoquinoline (**7**)

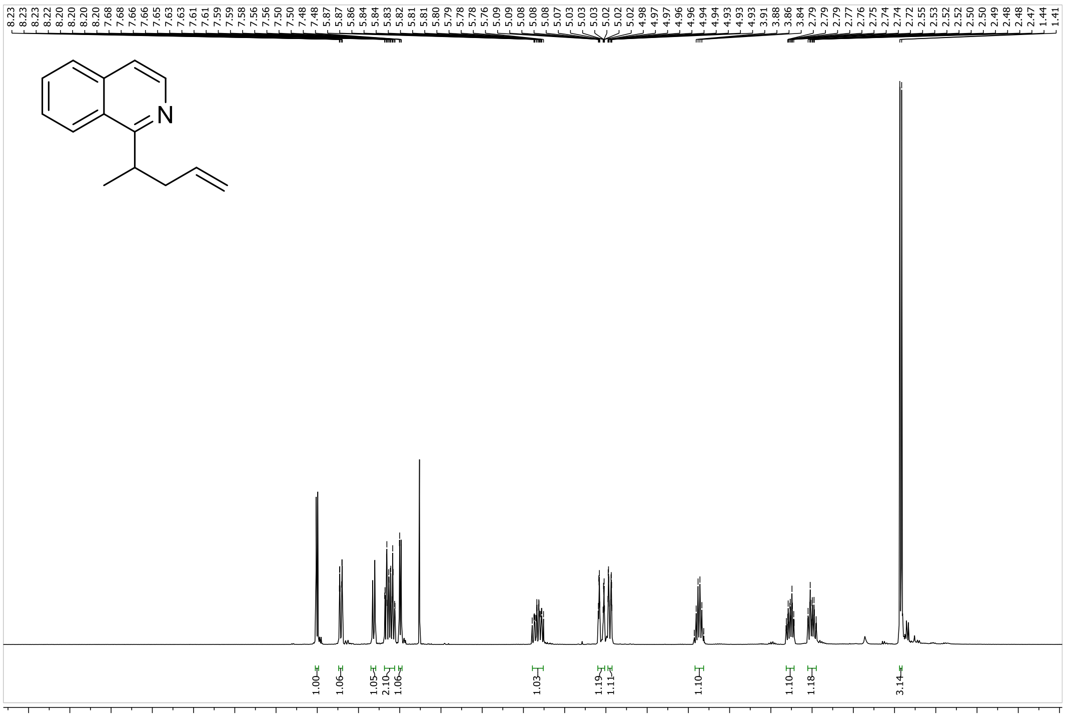

Figure S26. ^{1}H-NMR in $CDCl_3$ 1-(Pent-4-en-2-yl)isoquinoline (**8**)

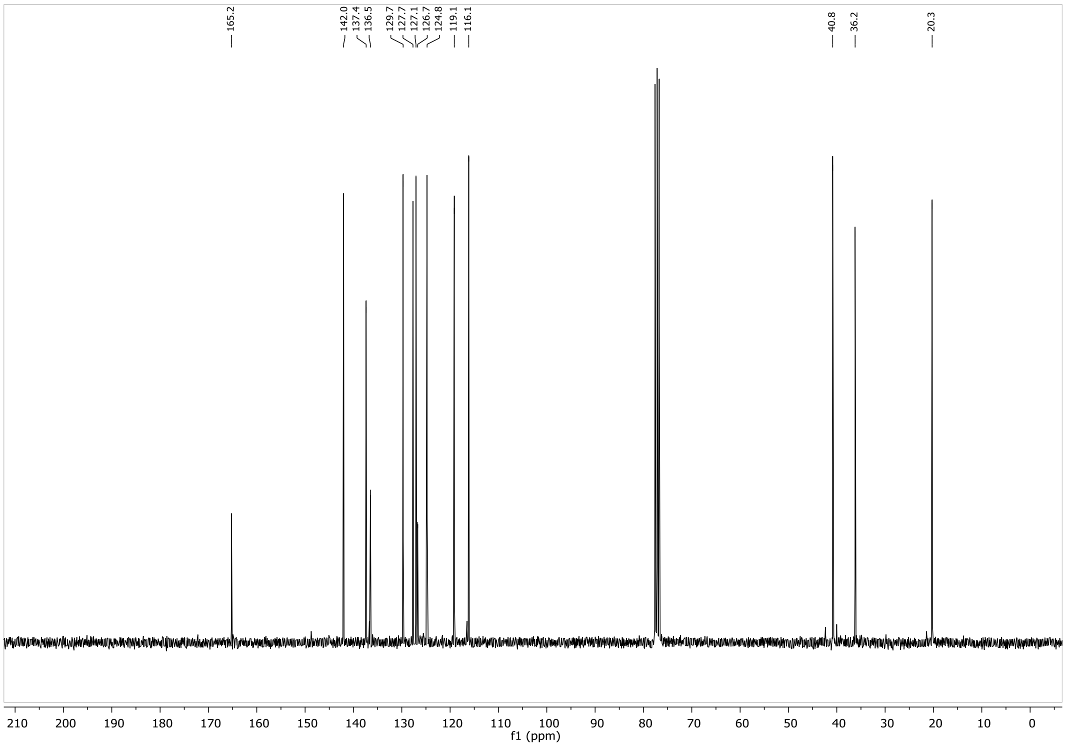

Figure S27. ^{13}C-NMR in $CDCl_3$ 1-(Pent-4-en-2-yl)isoquinoline (**8**)

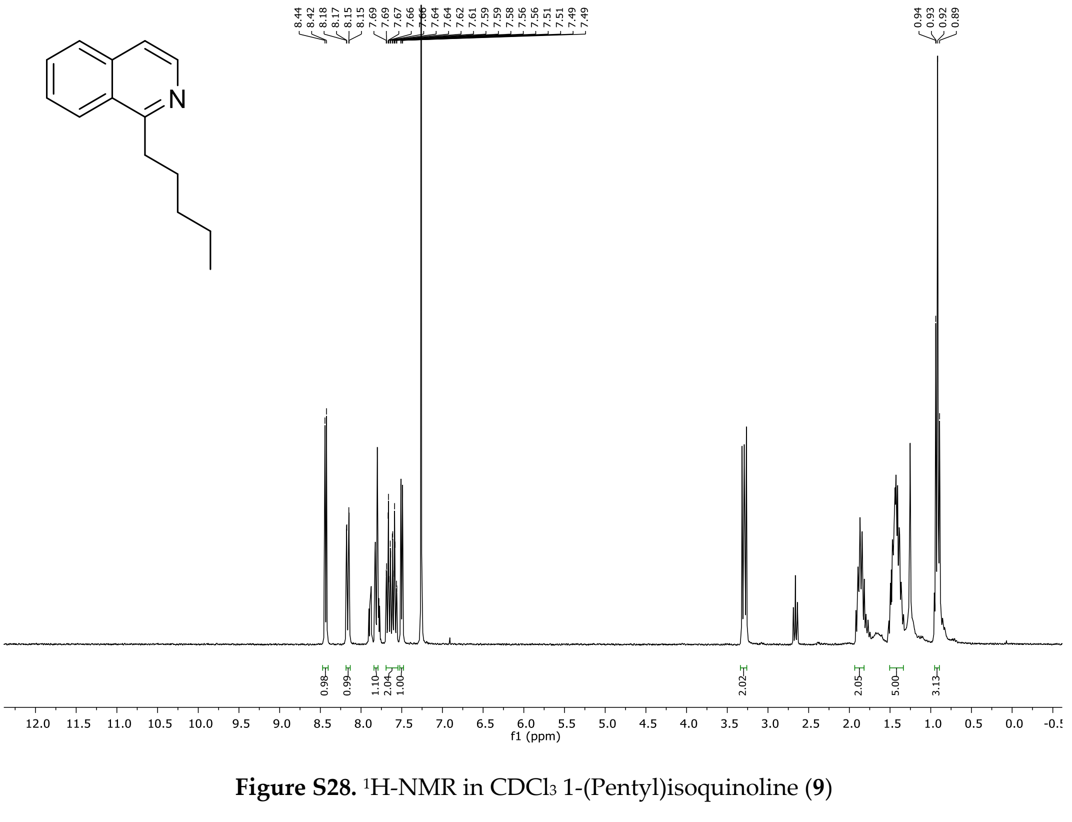

Figure S28. ^{1}H-NMR in $CDCl_3$ 1-(Pentyl)isoquinoline (**9**)

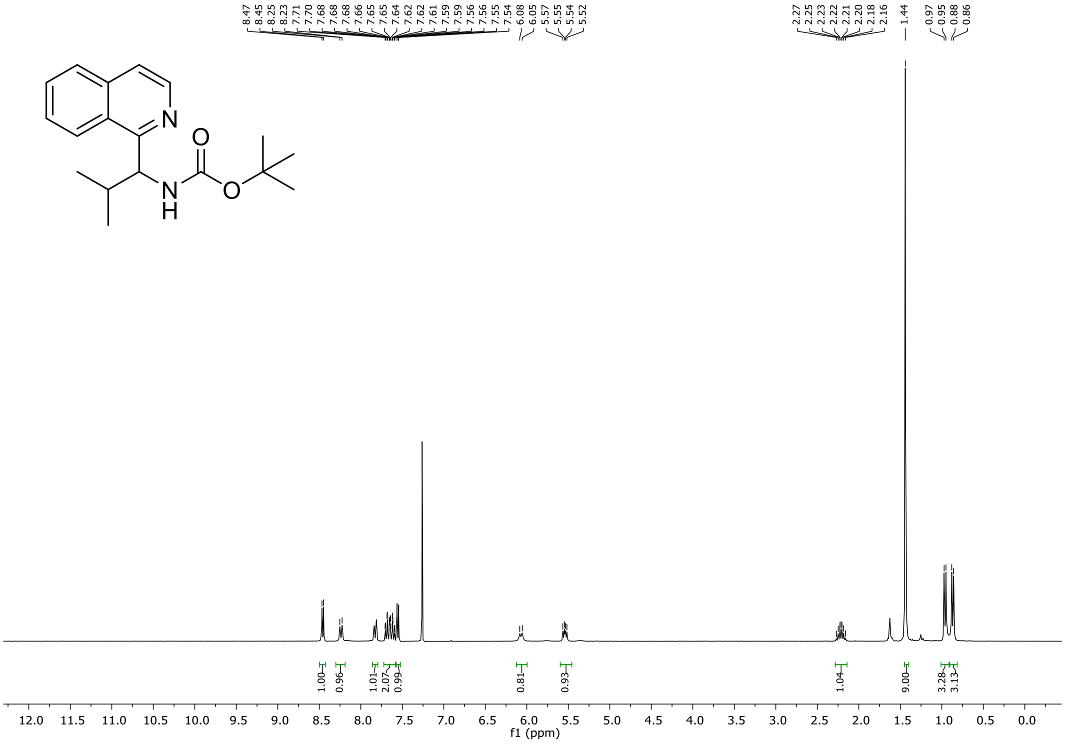

Figure S29. ^{1}H-NMR in $CDCl_3$ *tert*-Butyl-[1-(isoquinolin-2-yl)-2-methylpropyl]carbamate (**11**)

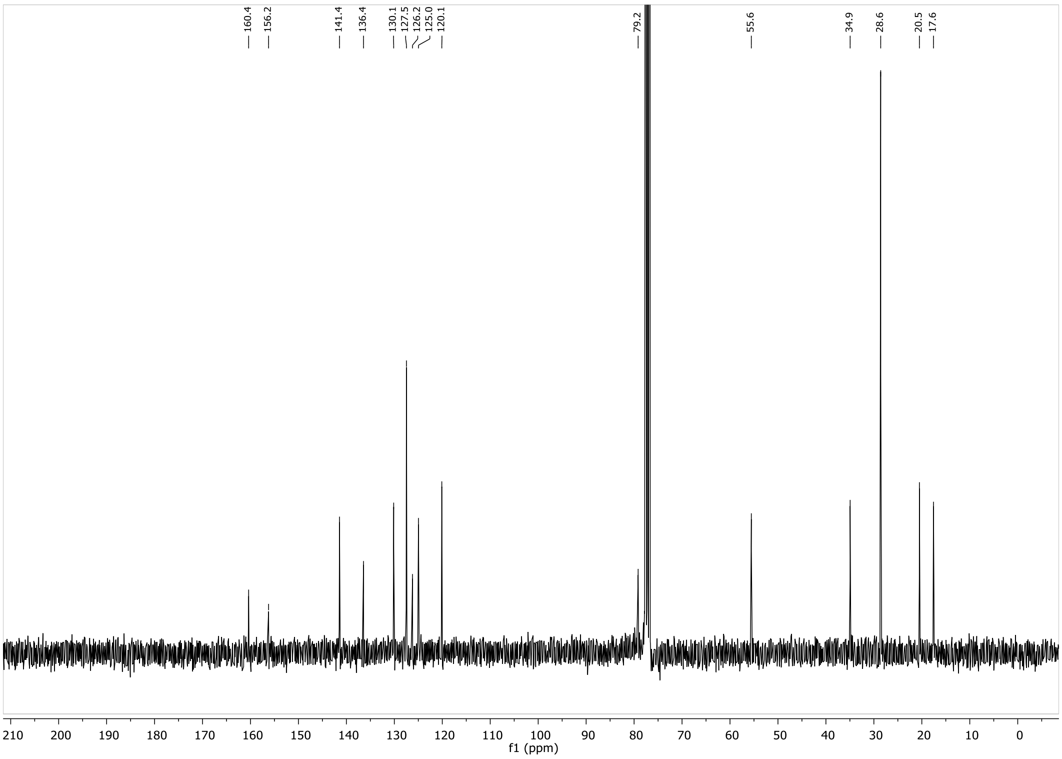

Figure S30. ^{13}C-NMR in $CDCl_3$ *tert*-Butyl-[1-(isoquinolin-2-yl)-2-methylpropyl]carbamate (**11**)

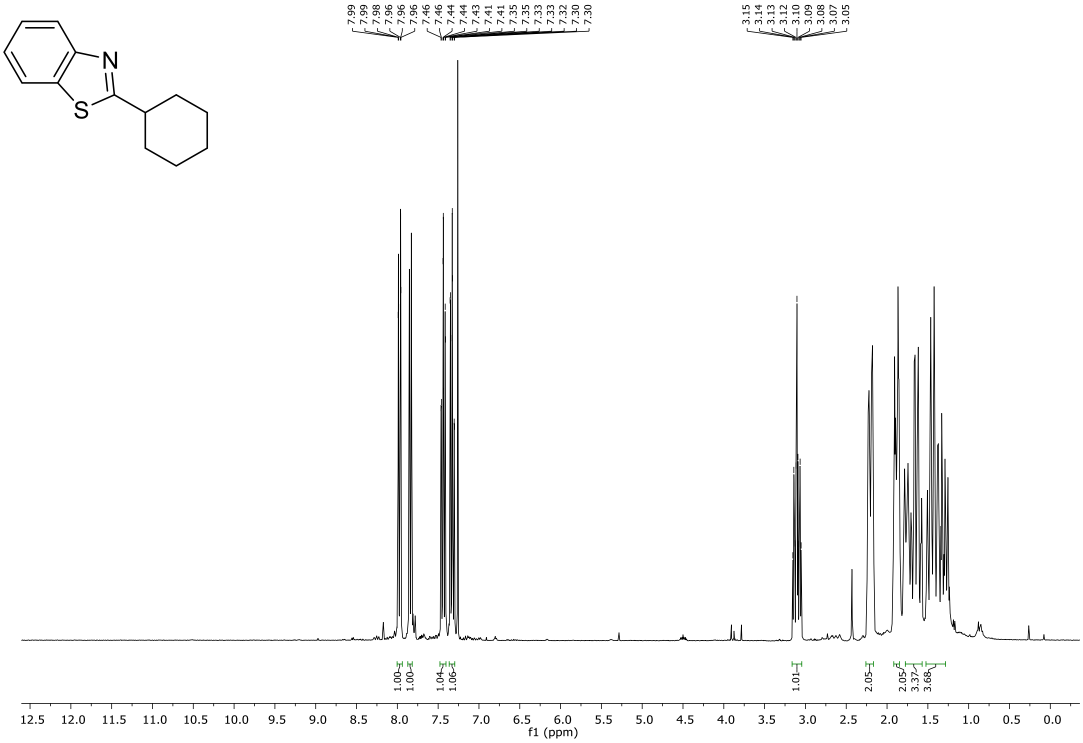

Figure S31. ^{1}H-NMR in $CDCl_3$ 2-Cyclohexyl-1,3-benzothiazole (**14**)

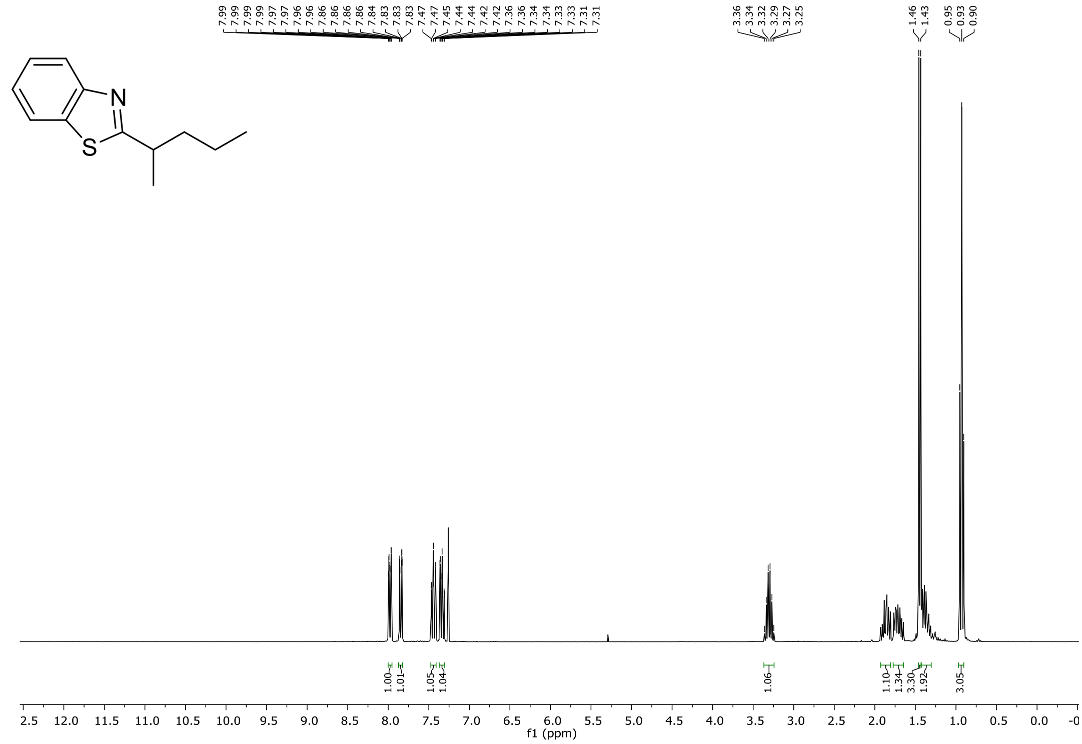

Figure S32. ^{1}H-NMR in $CDCl_3$ 2-(Pentan-2-yl)-1,3-benzothiazole (**15**)

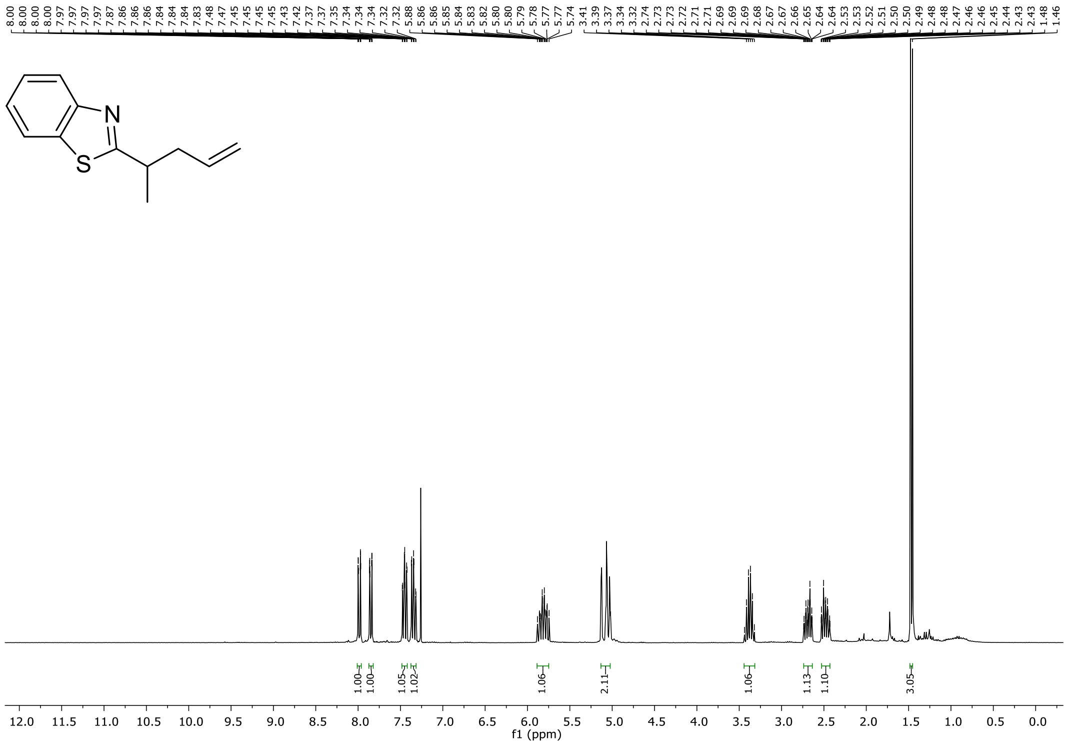

Figure S33. ^{1}H-NMR in $CDCl_3$ 2-(Pent-4-en-2-yl)-1,3-benzothiazole (**16**)

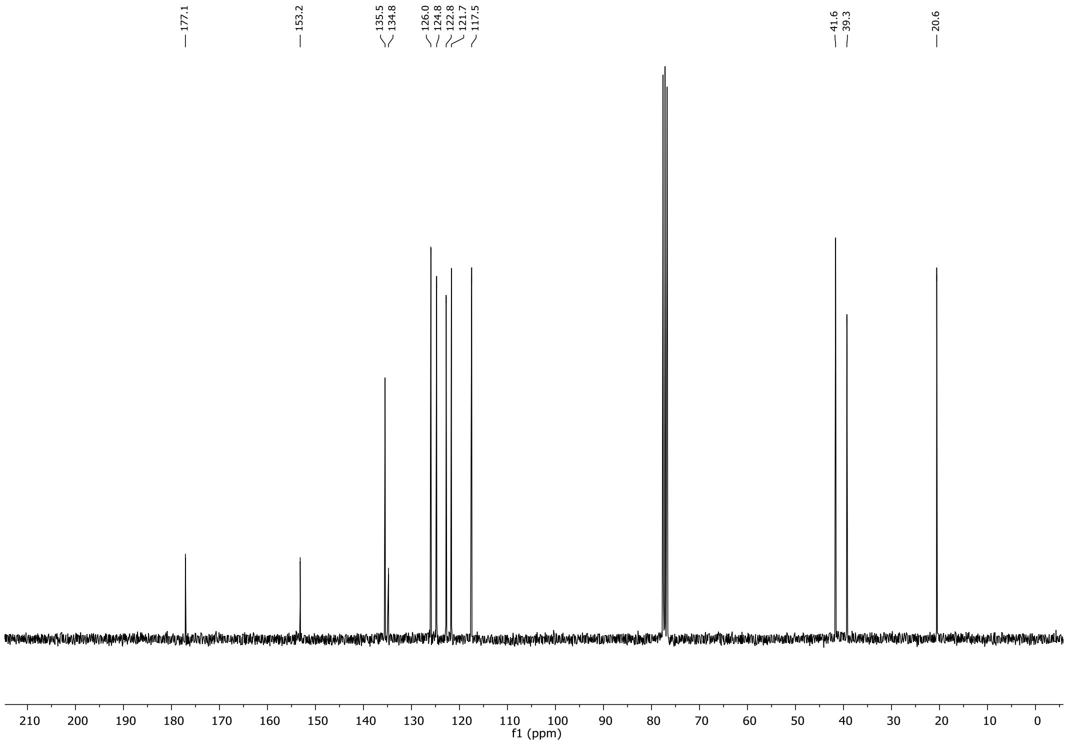

Figure S34. ^{13}C-NMR in $CDCl_3$ 2-(Pent-4-en-2-yl)-1,3-benzothiazole (**16**)

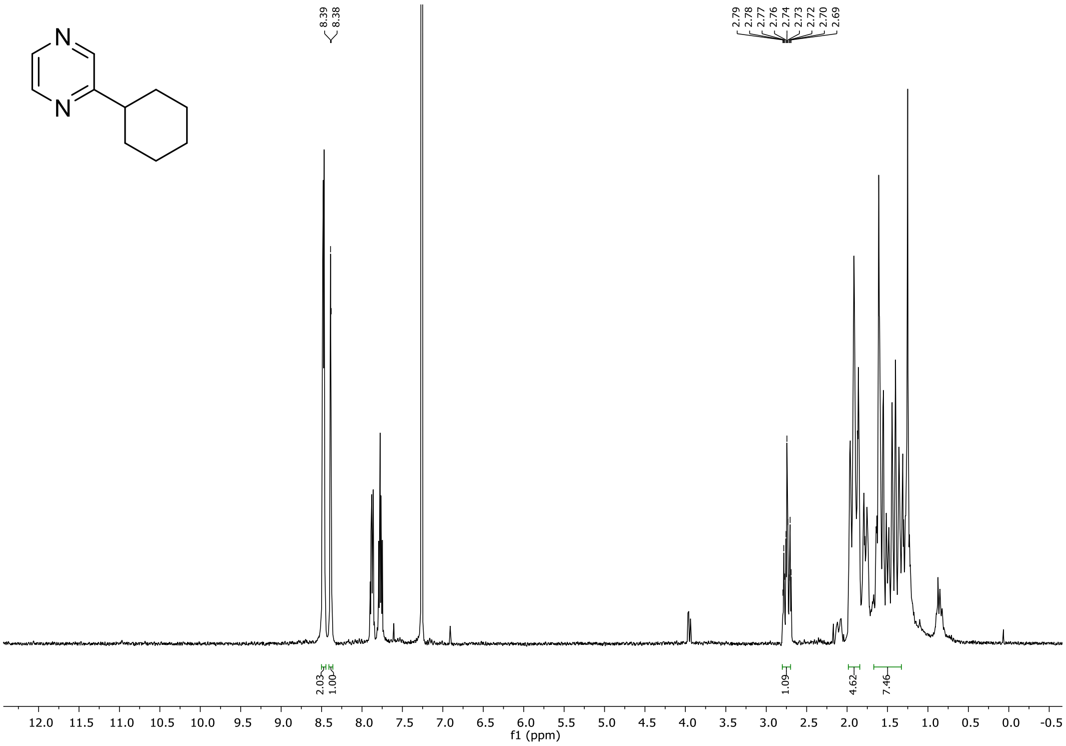

Figure S35. ^{1}H-NMR in $CDCl_3$ 2-(Cyclohexyl)-pyrazine (**17**)

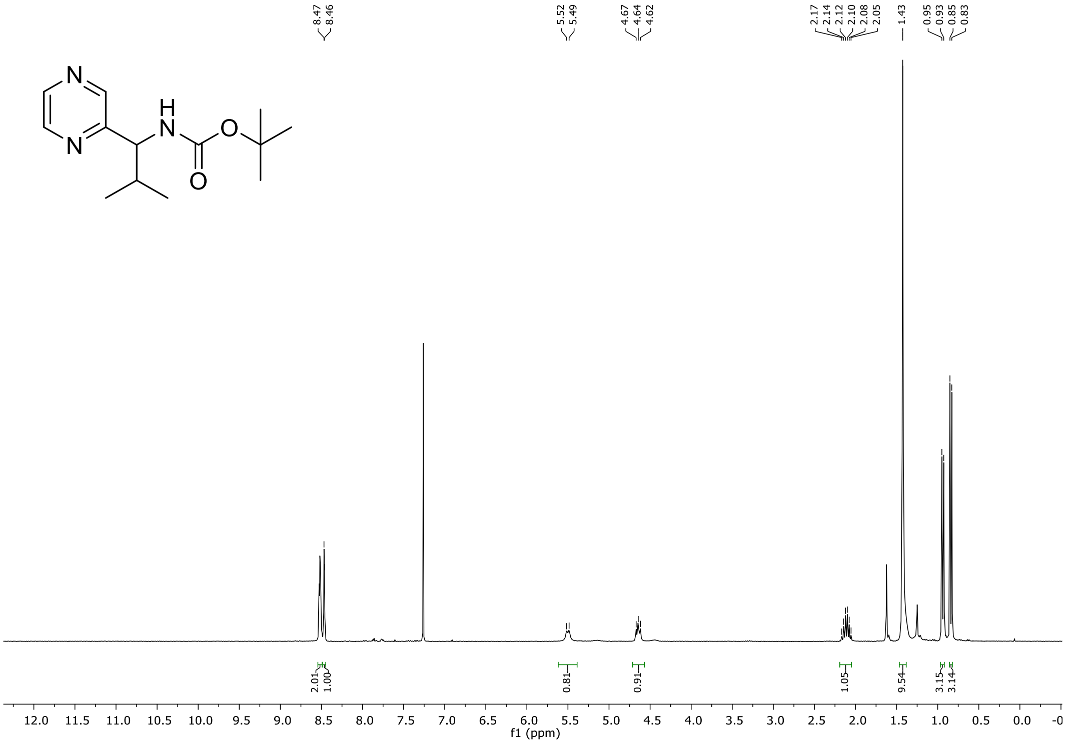

Figure S36. ^{1}H-NMR in $CDCl_3$ *tert*-Butyl-[2-methyl-1-(pyrazin-2-yl)propyl]carbamate (**18**)

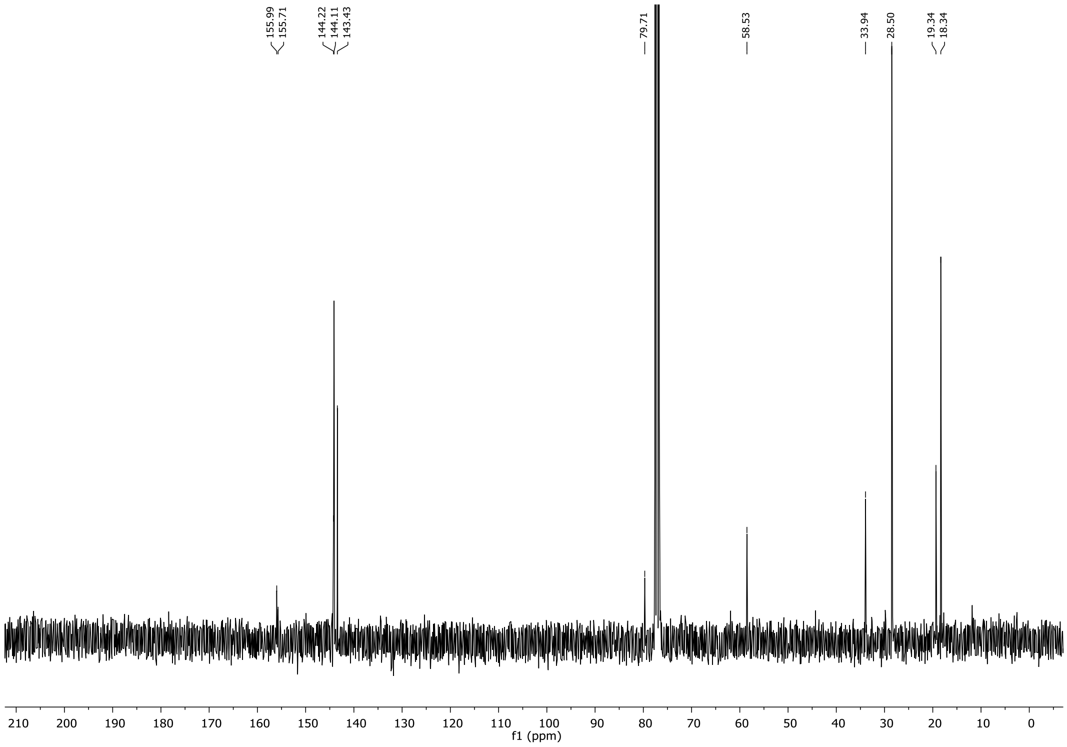

Figure S37. ^{13}C-NMR in $CDCl_3$ *tert*-Butyl-[2-methyl-1-(pyrazin-2-yl)propyl]carbamate (**18**)
